# Microhomology-mediated end joining induces hypermutagenesis at breakpoint junctions

**DOI:** 10.1371/journal.pgen.1006714

**Published:** 2017-04-18

**Authors:** Supriya Sinha, Fuyang Li, Diana Villarreal, Jae Hoon Shim, Suhyeon Yoon, Kyungjae Myung, Eun Yong Shim, Sang Eun Lee

**Affiliations:** 1 Department of Molecular Medicine, Institute of Biotechnology, University of Texas Health Science Center at San Antonio, San Antonio, TX, United States of America; 2 Children's Hospital of San Antonio, Baylor College of Medicine, San Antonio, TX, United States of America; 3 Korea Institute of Radiological & Medical Sciences (KIRAMS), 75 Nowon-ro, Nowon-gu, Seoul, Republic of Korea; 4 Center for Genomic Integrity, Institute for Basic Science, Ulsan 44818, Republic of Korea; 5 Department of Biological Science, School of Life Sciences, Ulsan National Institute of Science and Technology (UNIST), Ulsan 44818, Republic of Korea; 6 Department of Radiation Oncology, University of Texas Health Science Center at San Antonio, San Antonio, TX, United States of America; Columbia University, UNITED STATES

## Abstract

Microhomology (MH) flanking a DNA double-strand break (DSB) drives chromosomal rearrangements but its role in mutagenesis has not yet been analyzed. Here we determined the mutation frequency of a *URA3* reporter gene placed at multiple locations distal to a DSB, which is flanked by different sizes (15-, 18-, or 203-bp) of direct repeat sequences for efficient repair in budding yeast. Induction of a DSB accumulates mutations in the reporter gene situated up to 14-kb distal to the 15-bp MH, but more modestly to those carrying 18- and 203-bp or no homology. Increased mutagenesis in MH-mediated end joining (MMEJ) appears coupled to its slower repair kinetics and the extensive resection occurring at flanking DNA. Chromosomal translocations via MMEJ also elevate mutagenesis of the flanking DNA sequences 7.1 kb distal to the breakpoint junction as compared to those without MH. The results suggest that MMEJ could destabilize genomes by triggering structural alterations and increasing mutation burden.

## Author summary

Recurrent chromosome translocations juxtapose chromosomal fragments and alter expression of tumor suppressors or oncogenes at or near breakpoint junctions to develop distinct types of leukemias and childhood sarcomas. The prevalence of 2–20 bp of imperfect overlapping sequences (a.k.a. microhomology [MH]) at the breakpoint junctions suggests the type of repair events joining two chromosomal fragments and the formation of oncogenic chromosomal translocations. In this study, we discovered that MH-mediated end joining (MMEJ) operates with kinetics markedly slower than other repair options. The slower kinetics leads to extensive resection and drives hypermutagenesis at sequences flanking the break site. We also found that MH-mediated chromosomal translocations accumulate mutations at sequences up to several kilobases distal to the breakpoint junction as compared to those without MH. Our results revealed that MH contributes to genetic instability by facilitating chromosomal translocations and increasing mutational load at the sequences flanking the breakpoints.

## Introduction

The presence of short stretches of overlapping sequence (microhomology, MH) is a frequent feature of pathogenic chromosomal translocation breakpoints in human cells and has been implicated in juxtaposing two DNA ends for the error-prone repair of DNA breaks in both yeast and vertebrates [1–3]. This so-called microhomology-mediated end joining (MMEJ) is genetically distinct from Ku-dependent classical end joining or homologous recombination and becomes a prominent repair option when conventional repair mechanisms become inactivated or unavailable. Accordingly, MMEJ is frequently regarded as a back-up to the canonical repair pathways although it is still operational in cells retaining other repair options and contributes to a wide range of cellular chromosome maintenance processes including telomere maintenance and programmed immune receptor gene rearrangements [4, 5].

MMEJ is a highly error prone pathway because it inevitably entails deletion of inter-MH sequences and one of the MHs. MMEJ is also prone to chromosomal rearrangements due in part to the loss of intra-chromosomal joining bias [6]. To initiate MMEJ, DNA ends should first be resected and the flanking MHs for annealing should form ssDNA [7–10]. DNA resection also triggers DNA damage-induced checkpoints and the association of the strand exchange protein (Rad51)-DNA complex with ssDNA to initiate the homology search during recombination [11–13]. Furthermore, the formation of ssDNA at DNA ends inhibits non-homologous end joining (NHEJ), committing cells to homologous recombination (HR) and the MMEJ pathway [14]. Enzymatically, DNA end resection in eukaryotic cells comprises two distinct stages: initial resection by the Mre11 complex and more extensive resection by Dna2/Blm (Sgs1 in yeast) and Exo1 [15–18]. MMEJ is thus deficient in *mre11*-deleted cells or those deleted for CtIP [7–9, 19–22], a protein associated with the Mre11 complex that regulates its nuclease activity. Furthermore, expression of hypomorphic *rfa1* mutants, one of the three subunits in the replication protein A (RPA) ssDNA binding complex in yeast, elevates the MMEJ frequency almost 350-fold and induces gross chromosomal rearrangements with MHs at the breakpoint junctions [23]. Resection and the formation of ssDNA are thus key steps in MMEJ and likely dictate the types of repair outcomes and chromosomal integrity upon DNA breakage.

Interestingly, emerging evidence suggests that ssDNA also triggers elevated mutagenesis because cells ultimately need to fill-in the gaps formed during double strand break (DSB) repair and restore the DNA duplex by the actions of an error prone translesion polymerase [24–26]. DSB repair thus represents a significant source of mutagenesis and fuels genome instability in mitotic cells. Together these observations prompted us to consider if MMEJ could contribute to mutagenesis especially at the breakpoints of chromosomal translocations because ssDNA represents an obligate intermediate for the process. Indeed, breakpoint junctions of complex copy number variants often contain MH and are associated with a high frequency of mis-sense and in-del types of mutations at the flanking DNA likely due to error prone repair synthesis [27–29]. We surmise that some of these junctions and mutagenesis might arise by MMEJ.

## Results

### MMEJ is highly mutagenic

**Fig 1:**
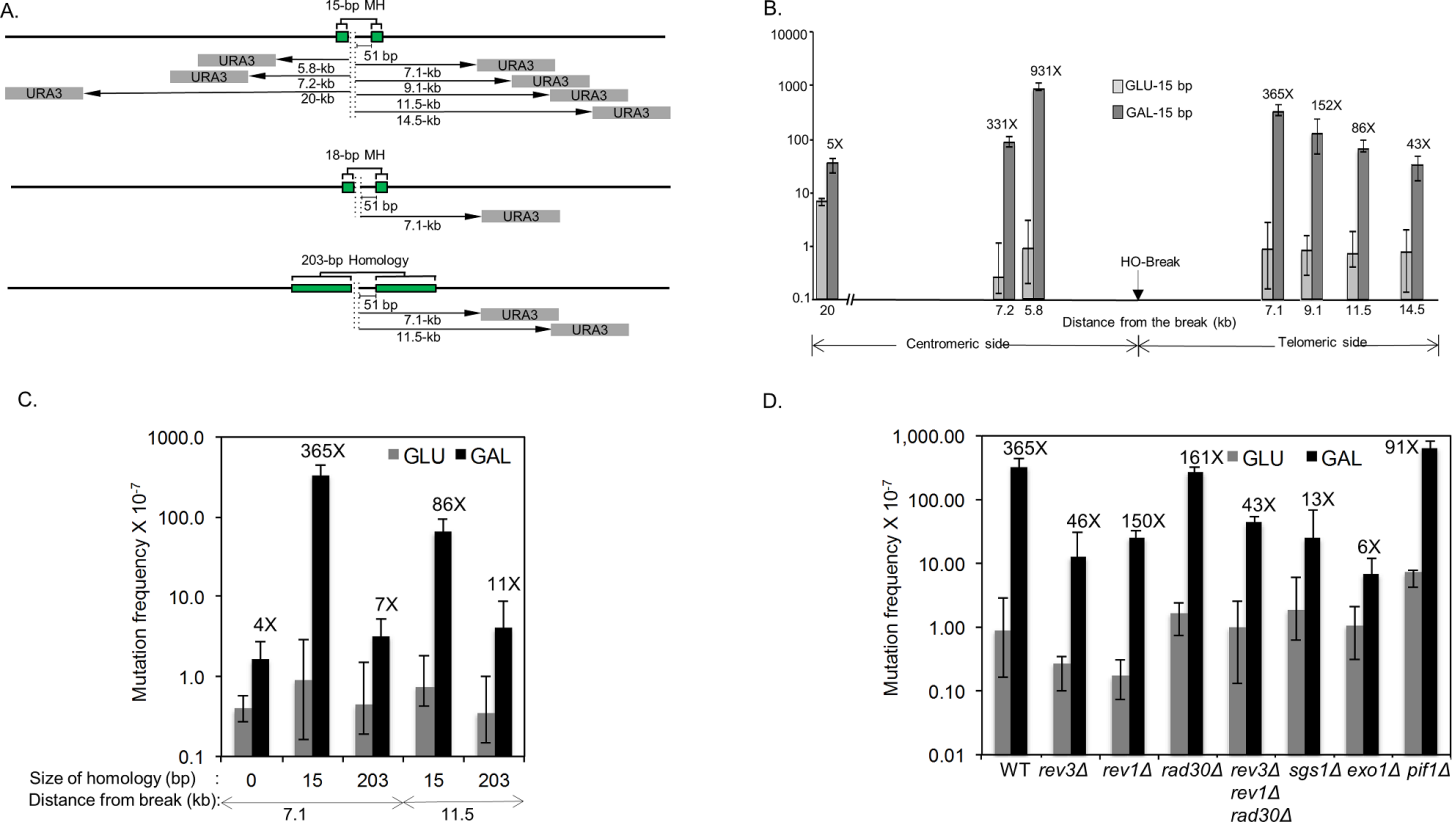
Microhomology-mediated repair induces hypermutagenesis. **A**. MMEJ and SSA systems. The position of 15- or 18-bp MH and 203-bp repeats flanking HO recognition sequences are shown as green boxes. The grey boxes indicate *URA3* reporter gene placed at several locations distal to an HO recognition site (5.8-, 7.1-, 7.2-, 9.1-, 11.5-, 14.5-, and 20-kb) from the break. The *HML*, *HMR* and *URA3* genes are deleted to avoid gene conversion events. **B.** DNA break-induced mutation frequency was calculated by the median of the fluctuation tests using the number of FOA^R^ survivors in yeast strains carrying the *URA3* reporter gene placed at indicated locations distal to the HO break site. The HO recognition site, shown by the arrow, is flanked by 15-bp MH that mediates MMEJ repair upon induction of galactose inducible HO endonuclease. Distance from the break and the fold stimulation, calculated by dividing the mutation frequency of induced cells (gal; galactose) by that of uninduced (glu; glucose) controls are shown below and above the bar graph, respectively. Plotted in the graphs are the median frequencies, 95% confidence intervals, and fold change. The values are also listed in S2 Table. **C**. The frequency of FOA^R^ survivors from yeast strains bearing 15-bp MH, 203-bp repeats or no homology flanking the HO break site, and the *URA3* reporter gene placed at 7.1- and 11.5-kb distal locations. The median frequencies, 95% confidence intervals, and fold change are shown. **D**. The frequency of FOA^R^ survivors in yeast strains with the indicated gene deletion was measured as described above. The median frequencies, 95% confidence intervals, and fold change are also listed in S2 Table.

To address if MMEJ is mutagenic, we set up a model MMEJ assay in yeast and placed a *URA3* reporter gene at several locations distal to an HO recognition site (5.8-, 7.1-, 7.2-, 9.1-, 11.5-, 14.5-, and 20-kb from the break, see Fig 1A). A DSB generated by HO cleavage is then flanked by 15-, 18- or 203-bp of direct repeat sequences 51-bp distal to the HO recognition site to mediate inter-repeat recombination (Fig 1A). The strain also lacks *HML* and *HMR*, two silent templates for gene conversion, and expresses HO endonuclease from the *GAL1/10* promoter integrated at the *ade3* genomic locus. The entire open reading frame of the endogenous *URA3* locus on chromosome V is also deleted to eliminate gene conversion between *ura3* sequences. Upon addition of galactose to the culture medium, HO is expressed (S1 Fig), and the resulting DSB is repaired by Rad52-dependent, but Rad51-independent single strand annealing (SSA) or MH-mediated events via annealing of flanking direct repeats (Fig 1A).

We measured overall survival frequency and FOA-resistant (FOA^R^) survival frequency by fluctuation tests, which reflect DSB repair and repair-induced mutation frequency, respectively (S1 and S2 Tables)[30]. We also measured mutation frequency in the *CAN1* gene located on the left arm of chromosome V as an internal control and used it to calculate the spontaneous mutation frequency intrinsic to cell proliferation and to determine the 95% confidence intervals (S3 Table).

We found that HO expression led to a nearly 10-fold reduction in survival in the strain with 15-bp repeats compared to that with 203-bp repeats, indicating that 15-bp repeats do not efficiently support DSB repair (S1 Table). HO expression led to an intermediate level of survival in the strain with 18-bp repeats as compared to those with 15- or 203-bp repeats. Induction of HO increased the mutation frequency of the reporter gene 5.1- to 931-fold in the strain with 15-bp repeats, 13.5-fold in the strain with 18-bp repeats, and 7.2-fold in 203-bp repeats (Fig 1, S2 Table). The highest mutation frequency was observed in the strain having the *URA3* gene inserted closest (5.8 kb) to the 15-bp repeats. The location of *URA3* did not have an impact on the survival frequency (near 8%) nor the frequency of *can1* mutations (Fig 1, S1–S3 Tables). The symmetry of the mutagenesis profile at either side of the break suggests that the distance to the repeats is one of the key factors dictating the frequency of mutagenesis (Fig 1B). Overall, the HO-induced mutation frequency was 51 times higher in cells that employed 15-bp of MH for repair (compare FOA^R^ frequency in the 7.1-kb telomere proximal location in 15-bp vs 203-bp repeat containing strains, Fig 1B), and the mutations were found at greater distal locations up to 14.5-kb from the break in MMEJ events compared to those in the 203-bp repeat strain (Fig 1B, S2 Table), suggesting that mutagenesis is inversely related to the repair frequency.

As a comparison, we also measured the mutation frequency before and after HO expression in strains lacking direct repeat sequences. The strains lacking repeats did not significantly (<4.1-fold) induce mutagenesis 7.1-kb distal to the DSB (Fig 1C, S2 Table).

To determine the types and spectra of mutations associated with DSB repair, *ura3* genes were recovered from FOA^R^ survivors and subjected to sequencing analysis (S2–S4 Figs, S4, S5 and S11–S16 Tables). Mutation spectra were analyzed in the *ura3* reporter on either side of the break to rule out the effect of chromatin landscape on the mutagenesis profile. We found that mutations were scattered throughout the open reading frame of the *URA3* gene but clustered to several hotspots with base substitutions/deletions at homo-polymeric runs. Surprisingly, we only detected two multiple mutants out of over 300 sequenced mutation events. The low frequency of widely spaced multiple mutations in the *URA3* reporter gene at DSB likely attributed to the small size of reporter gene (0.8-kb). G to C transversion-type events were dramatically elevated (42.4% without HO expression vs 83.3%, 60.9% or 64.4% after HO expression, see S4 Table) among mutations in the reporter placed at the 7.1 kb telomere-proximal location but not at the 5.8 kb centromere-proximal location after HO expression. We also observed minor differences in the mutagenesis patterns in strains with 15- or 203-bp repeats; for instance, recombination between 203-bp repeats induced far fewer base substitution type mutations at adenine relative to 15-bp MH or no homology repair events (p = 0.0549; S2–S4 Figs, S4 Table). The results suggest that MH-mediated repair is a powerful source of mutagenesis even for sequences that are tens of kilobases away from the break site.

### MH-mediated repair is kinetically slower than single strand annealing

**Fig 2:**
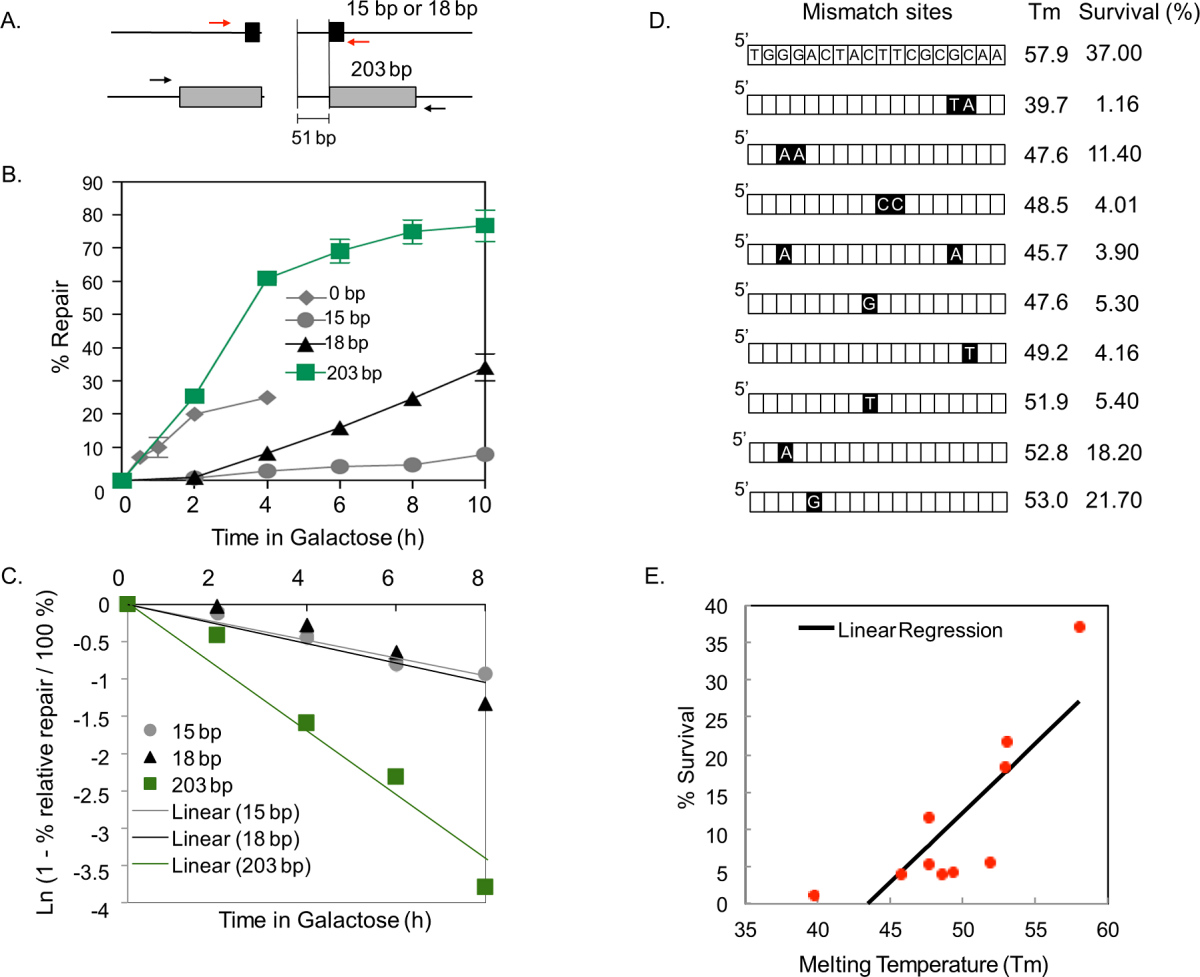
Kinetics of MH-mediated repair. **A**. Strategy to assess DSB repair kinetics. The black and grey boxes represent 15- or 18-bp microhomology (MH) and 203 bp homology flanking the HO-break site, respectively. The level of repair product was determined by quantitative real time PCR of genomic DNA isolated from an aliquot of cell culture after HO induction using primers flanking the repeats (red and black arrows). **B**. Graph showing the amount of repair products by annealing 203-bp homology or 0-, 15-, and 18-bp MH at indicated time points after HO-endonuclease induction. The results are the average of three independent experiments ± s.d. **C**. First order reaction kinetics of MMEJ products as a function of time post-HO expression. The slope represents the rate constant (k), which is constant regardless of MH sizes but is different in SSA between 203-bp repeats. **D**. Illustration of yeast strains with imperfect 18-bp MH. Black boxes indicate the position of base mismatches. The melting temperature (Tm) of each MH sequence and the percentage survival upon HO endonuclease induction are shown. Percent survival was calculated by dividing the number of colonies on YEP-galactose by the number of colonies on YEP-dextrose and multiplied by 100. **E**. Linear regression analysis of percentage survival vs. melting temperature (Tm) of strains carrying MH with one or more base mismatches. Percentage survival was positively correlated with Tm (p<0.05, R^2^ 0.667445).

To elucidate the basis for elevated mutagenesis in MH-mediated repair, we determined the timing of repair product formation by polymerase chain reaction (PCR) using primers flanking the repeats (Fig 2A, red and black arrows). To restrict our measurements of repair kinetics to a single cell cycle, we treated cells with nocodazole either prior to (S6A and S6B Fig) or just after (Fig 2B, S6C Fig) HO expression and rendered cells arrested at the G2 phase of the cell cycle. The cell cycle profile was confirmed by flow cytometry (S5 Fig).

We discovered that 15- and 203-bp repeat mediated repair events operate with distinctly different temporal kinetics in both conditions regardless of the order of nocodazole treatment and HO expression: SSA products using 203-bp direct repeats emerged at 2–4 h post-HO expression whereas the MH-mediated repair products were initially detected at 2 h but slowly accumulate up to 6–8 h post-HO expression (Fig 2B and 2C). These results further support the inefficiency of MH-mediated repair events.

We surmise that the protracted MMEJ kinetics reflects the instability of MH annealing and therefore, is inherent to the MMEJ process. Indeed, the rate of repair is higher between longer MH (18-bp) repeats than for shorter ones (15-bp), and the reaction follows first order kinetics (Fig 2C). To further test this possibility, we monitored MMEJ frequency in strains carrying MH of different melting temperatures by incorporating one or more base mismatches within the 18-bp repeats, thereby reducing the stability of MH pairing (Fig 2D). We discovered that the frequency of MMEJ was proportional to the melting temperature of flanking MHs (Fig 2D and 2E), supporting the premise that the stability of MH dictates the MMEJ frequency and corresponds to a key parameter of successful repair by MMEJ. Interestingly, the position of the mismatch also impinged on the MH-mediated repair frequency such that the mismatches towards telomere-proximal or central locations more severely disrupt MH-mediated repair (Fig 2D and 2E).

### Extensive resection occurs in repair via MH

**Fig 3:**
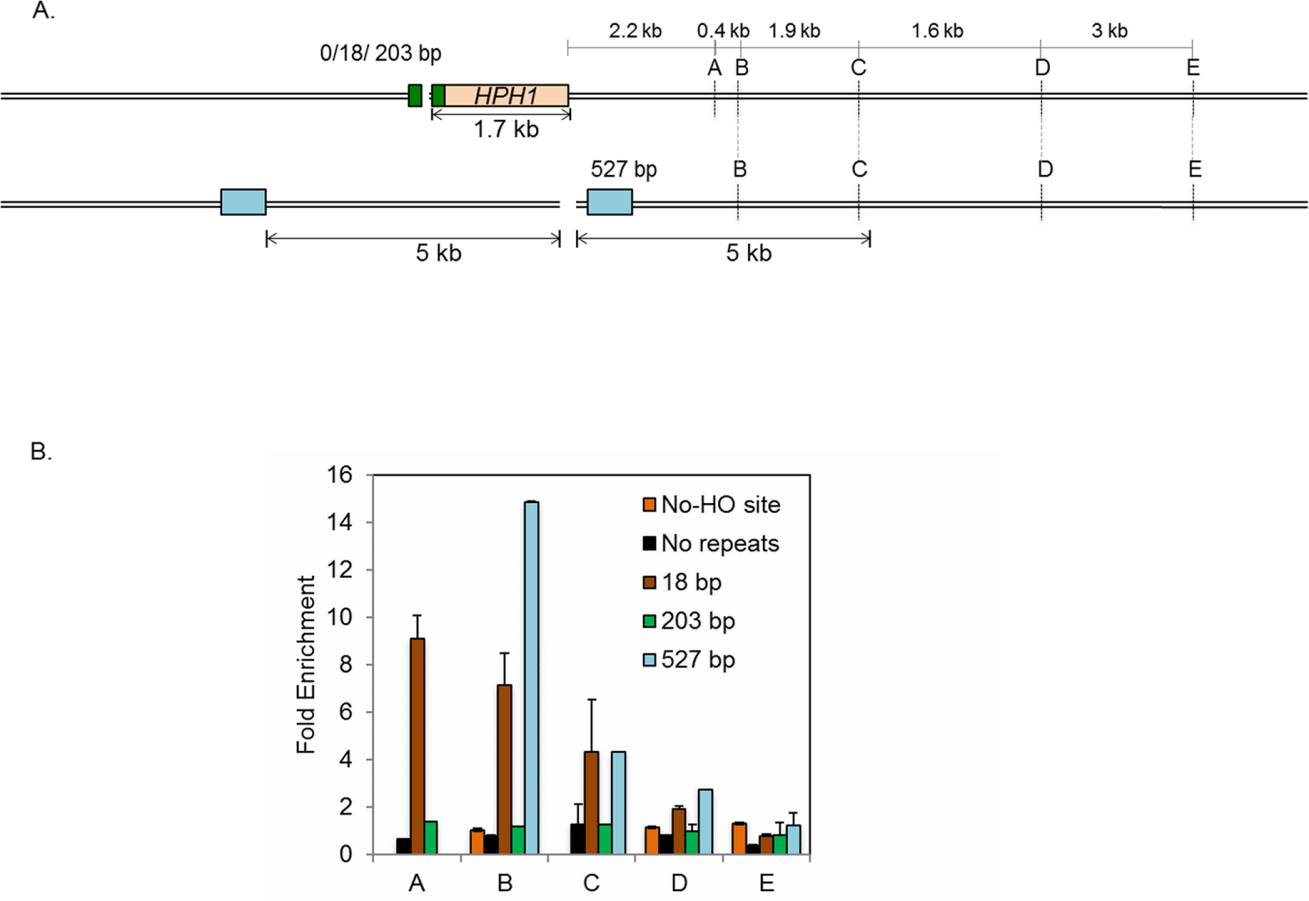
Extensive resection in MH-mediated repair. **A**. Diagram demonstrating the strategy to measure the amount of DNA repair synthesis during MMEJ and SSA repair as a proxy for end resection. The HO-recognition site and the flanking MH or homologies (18-, 203- and 527-bp; black boxes) at various locations distal to the break trigger MMEJ or SSA. The locations of primers (A, B, C, D and E, 2.2-, 2.6-, 4.5-, 6.1- and 9.1-kb from HO break site) to detect BrdU incorporation by chromatin immunoprecipitation using anti-BrdU antibody in a strain with 527 bp repeat are shown. Strains with 0-, 18- and 203-bp MH or homologies have a 1.76-kb *HPH* gene incorporated 51 bp proximal to the HO recognition site. In these strains the total amount of resection is calculated by adding 1.76 kb (size of the *HPH* gene) to the distance of the primers from the HO recognition site. The distance between homologies and the HO recognition site and the extent of resection required to uncover homologies in strain with 527 bp homology are also included. **B**. Fold enrichment of BrdU incorporation at A, B, C and D locations in strains carrying no MH, 18-bp MH, 203-bp or 527-bp repeats was calculated by measuring the amount of BrdU incorporation after HO endonuclease induction divided by that under no-HO conditions as described in Materials and Methods. The results are the average of three independent experiments ± s.d.

Evidence suggests that the amount of resection is directly proportional to the time needed for the repair [31]. The slow kinetics of repair events using MH might be accompanied by extensive resection at flanking DNA sequences. We therefore measured the extent of resection in both SSA and MH-mediated repair events. To date, most resection assays measured the amount of ssDNA in donorless yeast cells that lack all efficient repair options except limited end joining events [15, 32, 33]. However, in cells where resection leads to successful repair, the amount of ssDNA corresponds to the sum of resection and repair synthesis, complicating the accurate measurement of the extent of resection. To determine the amount of end resection in MMEJ events, we instead measured the amount of new DNA synthesis because the resected DNA should ultimately be re-synthesized by repair synthesis (Fig 3A). To detect the amount of repair synthesis, we labeled newly synthesized DNA using a nucleoside analog, bromodeoxyuridine (BrdU) in strains expressing both a nucleoside kinase as well as an equilibrative nucleoside transporter [34].

The amount of new DNA synthesis (i.e. resection) was monitored by incubating nocodazole-arrested G2 cells carrying 18-bp MHs in medium containing BrdU, which incorporates into nascent DNA during repair synthesis upon HO expression. Genomic DNA isolated from cells at several time points post-HO expression was pulled down with anti-BrdU antibody and analyzed by qPCR using a series of primer sets that anneal to the regions flanking the DNA break (Fig 3A). We found that BrdU incorporation extended up to 7.8-kb distal to the nearest repeats (location C) in MH-mediated repair (Fig 3B). The results were in stark contrast to SSA wherein the incorporation was not detectable even at 3.9-kb from the proximal repeat (Fig 3B, green bars). BrdU incorporation was not detected in strains lacking an HO cleavage site or in those without repeats (Fig 3B, orange bars and black bars, respectively). To further examine the extent of resection (and re-synthesis) during SSA, we constructed a strain in which the 527-bp repeat is situated asymmetrically at 5-kb distal and 0.5-kb proximal to the break site (Fig 3B, blue bars). In this strain, at least 5-kb of resection should occur to expose the requisite homology if resection proceeds symmetrically. Indeed, we found that BrdU incorporation is detected strongly at 3-kb proximal (location B) and up to 4.5-kb from the break (location C), but steeply declined at a site 6.1-kb proximal to the break (location D)(Fig 3B), indicating that resection is halted within a narrow zone about 1–2 kb beyond the repeat sequence. The results also suggest that the BrdU profile faithfully reflects the extent of resection and that MH-mediated repair events are accompanied by extensive DNA synthesis flanking the break site commensurate with the slow repair kinetics.

### Deficient resection reduces mutagenesis

**Fig 4:**
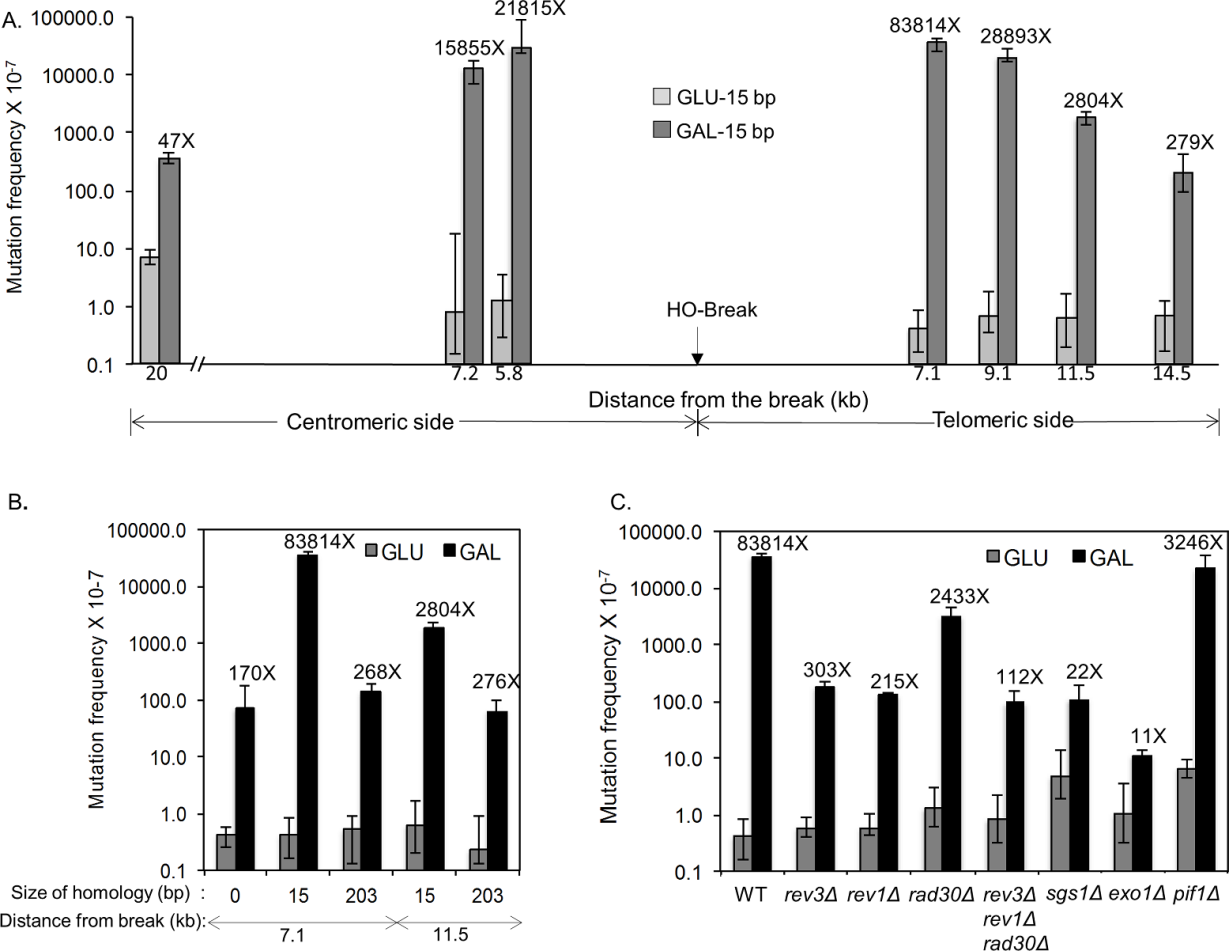
UV-induced mutagenesis during MMEJ repair. **A**. UV-induced mutation frequency was measured by scoring FOA^R^ survivors in yeast strains carrying the *URA3* reporter gene placed at indicated locations distal to the HO break site as described in Fig 1. The HO recognition site, shown by the arrow, is flanked by 15-bp MH that mediates MMEJ repair upon galactose induction of HO endonuclease. The distance from the break and the fold stimulation by DSB induction are shown below and above each bar graph, respectively. The median frequencies, 95% confidence intervals, and fold change are also listed in S6 Table. **B**. The frequency of UV induced FOA^R^ survivors from yeast strains bearing 15-bp MH, 203-bp repeat or no homology flanking the HO break site and the *URA3* reporter genes at placed at 7.1- and 11.5-kb distal locations. The median frequencies, 95% confidence intervals, and fold change are shown as in S6 Table. **C**. The frequency of UV-induced FOA^R^ survivors was measured as described in Fig 1 in yeast strains with the indicated gene deletions and bearing 15-bp MH flanking the HO break and the *URA3* reporter gene at the 7.1-kb distal location. The median frequencies, 95% confidence intervals, and fold change are also listed in S6 Table.

Emerging evidence suggests that ssDNA engenders elevated spontaneous and UV-induced mutagenesis [24–26, 35, 36]. According to this finding, mutation frequency may be directly proportional to the amount of end resection at given chromatin locations [25, 26], which could explain elevated mutagenesis in MH-mediated repair. Indeed, we found that UV treatment led to a dramatic (83,814-fold) increase in FOA^R^ (and thus Ura3-) frequency among survivors after HO expression when the *URA3* gene was inserted at 7.1-kb distal to the break site, and a moderate increase (279-fold) at 14.5-kb distal to the break site in a strain carrying flanking 15-bp MH (Fig 4A, S6 Table). In contrast, UV irradiation increased the frequency of FOA^R^ survivors when the *URA3* gene was inserted 7.1-kb (268-fold) or 11.5-kb (276-fold) distal to the break in long repeat strains (Fig 4B, S6 Table). As predicted, strong strand bias toward base substitutions at pyrimidines of the unresected strand was detected in the mutation spectra of the reporter placed at either side of the break (pyrimidine:purine = 31:2 and 22:6 at 5.8-kb centromere-proximal and 7.1 kb telomere-proximal to the break site, respectively) after UV and HO induction (S7 and S8 Figs, S5, S8 and S17–21 Tables). The results were consistent with the BrdU incorporation profile obtained from the ChIP assay that showed resection and repair synthesis reached at least 7.8-kb from the break site in MH-mediated repair but not in SSA (see Fig 3B).

In yeast, resection proceeds by two distinct stages: short range resection by the Mre11 complex, and long-range resection by Exo1 or Sgs1/Dna2 [13, 15, 17]. To test if resection and the formation of ssDNA trigger elevated mutagenesis flanking a DSB, we deleted *EXO1* or *SGS1*, two enzymes responsible for different resection pathways, and measured the mutation frequency of a *URA3* reporter gene at 7.1-kb distal to the break site upon HO induction. Should resection underlie elevated mutagenesis, deletion of *EXO1* or *SGS1* should reduce the mutation frequency upon HO expression in a strain carrying 15-bp MH. Indeed, the mutation frequency was reduced to 22- to 11-fold in *sgs1* or *exo1* deletion cells, respectively (Fig 4C). This was true even without UV irradiation (Fig 1D). We also found that break-induced mutagenesis depends on the Rev1 and Rev3 error-prone polymerases [37], but only moderately on Pif1 [38] and Rad30, suggesting that bubble migration as seen in break-induced replication is not chiefly responsible for the elevated mutagenesis in MH-mediated repair (Figs 1D and 4C)[39–41].

### MH induced elevated mutagenesis at chromosomal translocation breakpoints

**Fig 5:**
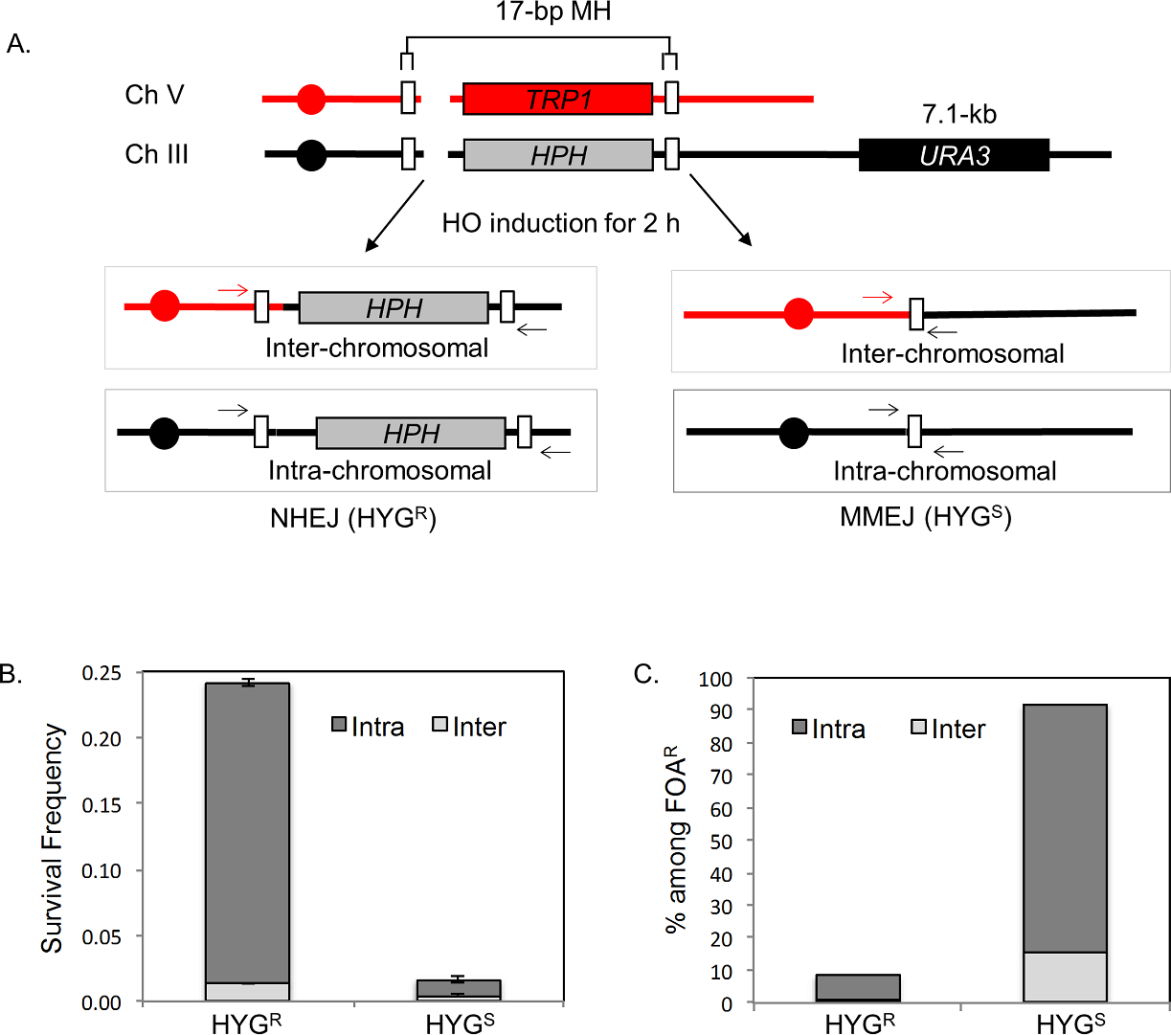
MH-induced mutagenesis at chromosomal translocation breakpoints. **A**. Schematics illustrating the yeast strain that produces intra- or inter-chromosomal MMEJ or NHEJ upon HO expression. The strain has two HO recognition sites, one on Chromosome III and the other on Chromosome V. White boxes denote the location of 17-bp MH near the break site. *HPH* and *TRP1* markers are shown. Four possible repair outcomes in this strain after DSB induction based on hygromycin sensitivity (HYG^s^) or resistance (HYG^r^) and the types of chromosomal joints are shown. The formation of chromosomal translocations was determined by PCR across the HO cleavage sites using primers annealed to two different chromosomes (arrows). **B**. Types of repair events among survivors. Survival frequency is calculated by dividing the number of survivors by the number of cells plated. A DSB was induced in the strain for 2 h by incubation in YEP-galactose and cells were plated on YEP-dextrose after serial dilution. The percentage of intra- and inter-chromosomal repair events was determined by PCR analysis of 100 colonies from each survivor. The results are the average of three independent experiments ± s.d. **C**. Types of FOA^R^ survivors after HO expression. The percentage of intra- vs inter-chromosomal repair events and the status of the hygromycin resistance gene are plotted. A DSB was induced for 2 h and cells were plated onto YEP-dextrose and subsequently replica plated onto 5-Fluoroorotic Acid (5-FOA) plates. 100 colonies from each experiment were analyzed by PCR to detect intra-chromosomal or inter-chromosomal repair products. The results are the average of three independent experiments.

Previously, we showed that flanking MH could trigger promiscuous end joining and chromosomal translocation if the repeats are placed in two different chromosomes [6]. To test if MH also induces hypermutagenesis at the regions flanking breakpoint junctions of chromosomal translocations, a yeast strain carrying two HO recognition sites, one at the *MAT* locus on chromosome III and the other at the *ura3* locus on chromosome V, was engineered to have 17-bp MH 2-kb telomere proximal to both HO cleavage sites (Fig 5A). The strain also contains a galactose-inducible HO endonuclease gene and lacks the *HML* and *HMR* loci so that the addition of galactose to the culture medium will induce DSBs at both cleavage sites but not elsewhere. DSB repair in this strain can proceed by intra- or inter-chromosomal MH-mediated repair and NHEJ. To distinguish the types of repair events, we have placed hygromycin resistance (*HPH*) and *TRP1* genes next to the HO cleavage site so MH-mediated repair will lead to hygromycin sensitivity and/or tryptophan auxotrophy. The formation of a chromosomal translocation was then determined by PCR across HO cleavage sites using primers that anneal to two different chromosomes (Fig 5A).

Upon inducing HO for only two hours, over 20% of cells survive, of which at least 90% repair one or both breaks by NHEJ (Fig 5B). The predominance of NHEJ events among survivors after 2 h of HO expression could be attributed to relatively faster kinetics of NHEJ compared to MH-mediated repair [10] and therefore, NHEJ likely acting before MMEJ for DSB repair. Faster NHEJ kinetics also limits the frequency of chromosomal translocations among survivors due to the inherent bias of NHEJ to intra-chromosomal joining [42]. In events where both ends are repaired by MH-mediated repair (i.e., *hph*^*−*^*trp1*^*–*^), no intra-chromosomal repair bias is found (S9C Fig). In contrast, most survivors from persistent HO expression arose from MH-mediated repair events (*hph*^*–*^), nearly half of which are chromosomal translocations, consistent with our previous observations (S9 Fig) [6].

To measure mutation frequency associated with MH-mediated chromosomal translocations, we inserted the *URA3* reporter at 7.1-kb distal to the *MAT* cleavage site and measured the frequency of FOA^R^ survivors after 2 h of HO expression. Induction of HO increased the FOA^R^ frequency 55.6-fold and most (91.8%) FOA^R^ survivors were hygromycin sensitive, indicating that MH-mediated repair events were significantly enriched among mutagenic repair (Fig 5C). The majority of mutations were few base pair indels or base substitutions at homopolymer runs as described in [25]. Notably, G to T transversions were increased 4-fold (p = 0.02) after DSB induction (S10 Fig, S9, S22 and S23 Tables). Strong enrichment (11-fold, p<0.001) of MH-mediated repair events (*hph-* events, confirmed by analyzing the repair junctions) among FOA^R^ survivors further confirms the high mutagenicity of such events as compared to those repaired by NHEJ (*hph+* events, confirmed by analyzing the repair junctions). Furthermore, among FOA^R^ survivors, chromosomal translocation events increased by 2.3-fold, with total survivors increasing from 6.8% to 16.1%. Together, these results suggest that MH-mediated chromosomal translocation could induce hypermutagenesis at sequences flanking the breakpoints.

## Discussion

Repair of a DNA double strand break (DSB) is frequently associated with elevated mutagenesis due in part to mutagenic repair synthesis that reverts accompanying ssDNA back to duplex form [24–26, 35, 36]. Indeed, hypermutagenesis was reported in ectopic gene conversion and break-induced replication even if mutagenesis is not always associated with error-prone translesion polymerases [35, 39, 40, 43]. Certain trinucleotide repeats, short palindromes, and interstitial telomeric sequences also induce chromosomal fragility and mutagenesis to flanking DNA sequences [44–48], likely because they trigger the formation of DNA DSBs and mutagenic DNA repair synthesis. We now show that MH-mediated end joining (MMEJ) can be added to the list of pathways endowed with extremely high mutagenesis potential, even up to tens of kilobases from the break site, underscoring its genome destabilizing capacity. Importantly, many of these mutations share features of clustered mutagenesis at or near chromosomal translocation breakpoints in human cancer cells [24, 26], raising the possibility that MMEJ contributes to some of these mutations. Alternatively, hypermutagenesis at the breakpoint junctions of MMEJ events reflects that yeast repair synthesis relies on error-prone polymerases, whereas in vertebrate cells repair is achieved by higher fidelity polymerases.

We propose that hypermutagenesis in MMEJ is linked with its slow kinetics. This is an inherent feature of MMEJ due to its reliance on the annealing of short MHs (S11 Fig), which itself is thermodynamically unstable and also counteracted by the presence of RPA [3, 6, 23]. The differences between MMEJ, HR and NHEJ with respect to kinetics as well as cell cycle dependency might dictate the order and timing of repair pathway choice for DNA lesions in cells and thus repair outcomes and associated mutagenesis upon DNA damage. It may also explain why MMEJ is more prominent when other competing and faster acting pathways become depleted or deficient [19, 49]. Alternatively, or in addition to MH annealing, other constraints such as degradation of antagonizing factors or the late recruitment of MMEJ components to the break site could contribute to slow MMEJ kinetics. Indeed, emerging evidence in vertebrate cells indicates that MMEJ could be blocked by a proteasome inhibitor [50] and normally confined to unique sub-nuclear compartments [51]. Additional studies are necessary to determine the underlying basis of slow MMEJ kinetics.

To analyze MMEJ-induced mutagenesis, we employed an experimental strategy that entails replica-plating of the surviving colonies after several divisions on non-selective medium following HO expression (see Materials and Methods for additional details). The arrangement was necessary because MMEJ is a slow repair process and 5-FOA kills yeast cells rapidly and does not allow residual divisions needed to establish DSB induced mutagenesis. Acute cell killing by FOA medium might also explain why UV irradiation did not increase spontaneous mutagenesis in *URA3* as compared to that measured *CAN1* mutagenesis using canavanine containing medium for selection (see S2, S3, S6 and S7 Tables). Mutation frequencies without DSB induction were measured by direct plating onto FOA containing medium. Excessive killing of recently formed *ura3* mutants after transfer to FOA containing medium could thus account for the apparent lack of mutagenesis upon UV irradiation. Nonetheless, our results fully establish that MMEJ is far more mutagenic than SSA and NHEJ, in which all mutation frequency measurements involved identical set-ups and the methodologies used.

The presence of MH in most pathogenic chromosome translocations and complex genome rearrangements highlights MH as a driver for genome destabilization via either variant end joining, HR, or template switch (TS) mechanisms [52]. Complex genome rearrangements (CGRs) and somatic rearrangements are also accompanied by dramatically high levels of mutagenesis of DNA sequences at or near breakpoints harboring MHs [27, 53]. Analysis of breakpoint junctions with single base-pair resolution from 95 tumor samples revealed that somatic rearrangements across all cancer cell types are frequently associated with hypermutagenesis up to 10-kb flanking the breakpoint junctions [29]. Most of these mutations are transversion types [28, 29]. These results raise an intriguing possibility that breakpoint mutagenesis could partially be attributed to MMEJ driven events. Specifically, mutations observed at locations far distal from the break site cannot readily be explained by current models of microhomology-mediated BIR or TS, yet are consistent with long-range mutagenesis in MMEJ [39, 52, 54].

Under experimental conditions, MMEJ and other repair events could sharply induce mutagenesis at DNA flanking DSBs. It raises the tantalizing possibility that break-induced mutagenesis could drive the progression of diseases and potentially dictate cellular responses to current treatment protocols. Mutations occurring at flanking DNA sequences could also offer a unique strategy to selectively target disease cells that harbor pathogenic chromosomal rearrangements using neighboring gene deficiency as additional biomarkers. However, mutagenesis might be confined to a small fraction of repair events and many of these mutations do not necessarily lead to gene deficiency. Nonetheless, it will be interesting to explore if MH at the breakpoint junctions impinges on the aggressiveness of diseases and/or the treatment outcomes and could thus be exploited to identify the best therapeutic approaches according to the types of repair events triggering chromosomal rearrangements.

## Materials and methods

### Strains

All yeast strains (S10 Table) are derivatives of JKM139 or JKM179 and were made by amplification of the hygromycin B phosphotransferase (*HPH)* gene from pAG26 with 90-bp oligonucleotides, containing 20-bp of homology to *HPH*, various sizes of microhomology/homology sequence, and homology to the Z1 region of *MAT*α/a on chromosome III. Briefly, the SS203 strain containing direct 203-bp repeats flanking the Z1 region of *MAT*α on chromosome III was constructed by the Golden Gate technique using primers ssa1, ssa2, ssa3 and ssa4 [55]. For SS527 strain construction, 527-bp fragments encompassing *MATα* Z2 sequence and *TAF2* 3’ end sequence were amplified (527-F and 527-R) and fused with the *HPH* gene at the 3’ end by PCR, and integrated into the *PHO87* gene locus by PCR-based gene targeting (primers to introduce homology for integration: Pho87-HYG-F and TAF2-3-R). Gene deletion mutants were constructed by a PCR-based technique using oligonucleotides flanked by terminal sequences homologous to the open reading frames of target genes. BrdU incorporating (BrdU-inc) strains were constructed by the one step integration method as described previously^**52**^.

### HO endonuclease induction

Yeast cells grown in YEP-glycerol media for 16 h were serially diluted and plated onto YEP-dextrose and YEP-galactose plates. Galactose induces HO endonuclease expression. Short or pulsed HO expression was achieved by adding 2% (w/v) galactose to the logarithmically growing yeast cells in YEP-glycerol medium, and after the indicated time of incubation, aliquots of culture were removed and plated onto YEP-dextrose to inhibit further HO endonuclease expression. Survival frequency was calculated by dividing the number of colonies on YEP-galactose by the number of colonies on YEP-dextrose plates.

### Quantitative PCR-based assay to detect MMEJ product formation in real time

Logarithmically growing yeast cells were incubated in YEP-glycerol for 16 h and 2% (w/v) galactose was added to the culture 2.5 hours prior to or after nocodazole (15 μg/ml) induced G2 cell cycle arrest. At different time points (0–10 hours), aliquots of culture were harvested and genomic DNA was isolated using the MasterPure Yeast DNA Purification Kit (Epicentre Biotechnologies). The amount of repair product was determined by quantitative PCR using primers flanking the newly re-joined DNA and normalized by amplification of a control locus in the genome (*YEN1* genomic locus). To eliminate uncut or NHEJ events, genomic DNA was digested with *Psi*I restriction enzyme prior to PCR analysis. The recognition sequence of *Psi*I is located in the inter-repeat DNA and is thus deleted in MMEJ products but not in NHEJ products.

### Detection of repair synthesis by BrdU-incorporation assay

A single colony of *S*. *cerevisiae* cells was inoculated in 2–3 mL YEP-dextrose and cultured for 12–24 h. One ml of cells was harvested, washed with YEP-glycerol, transferred to 200 ml YEP-glycerol, and cultured overnight. Nocodazole was added to the culture at a final concentration of 20 μg/ml, and cells were cultured for another 3 h (at this point, cells were examined under the microscope to ensure that >90% of cells are arrested at G2/M). A double strand break was induced by adding galactose at 2% final concentration, and BrdU was supplemented to the medium at 400 μg/ml. Cells were cultured for another 10- to 13-h (no repeats & 18-bp) or 4- to 6-h (203-bp & 527-bp), and then harvested and washed with 50 mM EDTA. Genomic DNA was isolated by standard glass bead-based DNA extraction. Isolated DNA was re-suspended in 200 μl TE supplemented with RNase A (100 ng/ml), incubated at 37°C for 1 h, and then sonicated to sheer the DNA to fragments ranging from 200 bp to 700 bp. DNA was separated by 1.2% agarose gel and fragments ranging from 200–700 bp were extracted using a gel purification kit (Qiagen). One μg of purified DNA fragments (20–50 μl), 10 μg ssDNA and 10 μl 10xPBS, supplemented with distilled H_2_O to a final volume of 100 μl, was mixed, pelleted in a microcentrifuge, and placed in a 100°C heat block for 10 min. The mixture was then supplemented with 400 μl PBST (PBS, 0.1% Triton X-100), and 1 μl anti-BrdU antibody, and incubated at 4°C with rotating for 2 hrs. Five μl of the reaction was taken as 1% input, and mixed with 200 μl elution buffer. IP reactions were supplemented with 30 μl Dyna magnetic protein G beads (Invitrogen), and incubated for another 2 h. DNA-antibody-protein G bead complexes were subjected to extensive washing as follows: 1) 1 ml lysis buffer (50 mM HEPES pH 7.5, 1 mM EDTA, 140 mM NaCl, 1% Triton X-100, 0.1% NaDoc) for 5 min, 3 times; 2) 1 ml high salt lysis buffer (50 mM HEPES pH 7.5, 1 mM EDTA, 500 mM NaCl, 1% Triton X-100, 0.1% NaDoc) for 5 min; 3) 1 ml washing buffer (100 mM Tris-HCl pH 8.0, 1 mM EDTA, 1% Triton X-100, 0.1% NaDoc) for 5 min; 4) TE (10 mM Tris-HCl, 1 mM EDTA) for 5 min. The supernatant was removed completely, and DNA-antibody complexes were eluted with 2 x 100 μl elution buffer (10 mM Tris-HCl, 1 mM EDTA, 1% SDS) by incubating the tube at 65°C for 15 min. Beads were precipitated by magnetic apparatus, DynaMag2, and the supernatant was transferred to a new tube. Eluted DNA-antibody complexes were supplemented with 10 μl glycogen, 25 μl 3 M NaAC (pH 5.6) and 500~750 μl ice cold ethanol and kept at -80°C for >2 hrs. DNA was precipitated by centrifugation at 13,000 rpm at 4 °C for 15 minutes. Precipitated DNA was resuspended in 300 μl distilled water and subjected to quantitative PCR analysis using a series of primer sets that anneal to the regions flanking the DNA break site.

### Mutagenesis associated with DSB repair

Logarithmically growing yeast cells were incubated in YEP-Glycerol medium for 16 h and then diluted with fresh 2% (w/v) galactose (Gal) synthetic complete media to induce Gal-HO-endonuclease expression for generation of site-specific DSBs. After 4 h of growth in galactose 10^8^ cells were spun down and plated onto 150 mm YEP-GAL plates. To test UV-induced mutagenesis, the YEP-GAL plates were subsequently irradiated with 20 J m^-2^ ultraviolet-C (UV-C) using a Stratalinker (Stratagene). UV-C treated and untreated cells on YEP-GAL plates were incubated at 30°C for 12 h and then replica plated onto media containing 1 mg/ml 5-fluoroorotic acid (5-FOA) and 60 mg/ml L-canavanine to select for *ura3* and *can1* mutants.

To measure the frequency of FOA-resistant colony formation, we used the replica plating of surviving colonies after short-term (12 h) growth on YEP-GAL instead of a more standard method that entails simply plating cells onto FOA-GAL plates. We opted for this strategy because MMEJ events proceed significantly slower than SSA or gene conversion events and such slow repair product formation could impinge on the rate of FOA resistant colony formation. Indeed, the standard plating method greatly underestimate (almost 89-fold lower) the FOA colony formation frequency in a strain with 15-bp repeats; in contrast, the values obtained from standard plating and replica plating methods are almost identical in SSA-induced mutagenesis. We concluded that measurement of mutation frequency by the standard plating method is not suitable for MMEJ-mediated mutagenesis analysis and far less accurate even if the replica plating method may lead to minor fluctuations. Importantly, the replica plating method used here is remarkably reproducible with only <20% fluctuation between different trials (3 independent trials).

In order to determine the level of induced mutations, we calculated “mutation frequency” with r/N (‘r’: the total number of mutants, ‘N’: the total number of cells plated). Since we scored mutation events that are induced by an HO break, and formed within a single cell cycle, the frequency should be more appropriate in this case. The assay measures the frequency of 5-FOA resistant colony formation per viable cells. For the statistical interpretation of the data, the web tool “FALCOR” was used to calculate confidence intervals about the median with the cumulative binomial distribution of the rank value of M [30]. Significance testing was done via the Mann–Whitney U test [56] using the FALCOR program. The binomial distribution function used to calculate 95% confidence intervals is: Pr (probability) = n!/k!(n-k)! x (0.5)^n^; n = number of cultures in the experiments, k = the rank value.

The ‘‘No-GAL”control cells (10^8^) were plated on Media containing 5-fluoroorotic acid (5-FOA) and L-canavanine to measure the spontaneous mutation frequency. For an additional control, mutation frequency in a “no homology” strain was also measured. Continuous Gal-induced HO endonuclease expression led to only 0.1% survival in this strain; therefore, for accurate mutation frequency measurements, the “no homology” strains were treated with galactose in order to induce endonuclease for only 2 h. Otherwise, all strains were treated identically. For further analysis of type of mutation pattern, a single FOA^R^ mutant was recovered from each culture to avoid scoring of redundant mutations arising from the same mutated parent, the *URA3* reporter was amplified by polymerase chain reaction (PCR) using primers annealing upstream and downstream of the gene, and products were sent (Beckman Coulter) for single pass sequencing using multiple primer sets. The primer sequences and additional information are listed in the “Primer List” (Table 1).

**Table 1:** Primer list.

Strain	Primer	Sequence
MH15	mh15-5'	CGGAATATGGGACTACTTCGCGCAACAGTATAATTTTATAAACCCTGGTTTTGGTTTTGTAGAGTGGTTGACGAGACTACTTCGCGCAAGGAAGCTTCGTACGCTGCAGG
	mh15-3'	ACACTCTATAAGGCCAAATGTACAAACACATCTTCCCAATATCCGTCACCACGTACTTCAGCATAATTATTAGGCCACTAGTGGATCTGA
M18	m18-5'	AATATGGGACTACTTCGCGCAACAGTATAATTTTATAAACCCTGGTTTTGGTTTTGTAGAGTGGTTGACGATGGGACTACTTCGCGCAAGGAAGCTTCGTACGCTGCAGG
	m18-3'	ACACTCTATAAGGCCAAATGTACAAACACATCTTCCCAATATCCGTCACCACGTACTTCAGCATAATTATTAGGCCACTAGTGGATCTGA
SS2	ssa1	GACGCTCTTCACCGCCCACTTCTAAGCTGATTTCAATCTC
	ssa2	GACGCTCTTCTACTCGTCAACCACTCTACAAAACC
	ssa3	GACGCTCTTCTAGTCTGGAAGTCAAAATACTCAGTTTC
	ssa4	GACGCTCTTCCATGCCGTCGACCTGCAGCGTACGAAGCTTCCGCGCGAAGTAGTCCCATATTCCGTG
SS527	527-F	TCAGTATAGCTATCCTATTTGA
	527-R	GGTAAACTTGATATTGTTATAAAG
	Pho87-HYG-F	AATTGGCTTAGGCAATGGATACTTGAAATATTCAATTTGAAGCTTTTCTTGCATAAGTTTCTCAGCTTCATCTTCAATAGGAAGCTTCGTACGCTGCAGG
	TAF2-3-R	GTCGTACCTGCACGACCACATTGTTTGGGAAAGAAGCAACACATGGAAAGACATGTTGGAGCGCCTCTCTTCGCAAAATAACGGGTAAACTTGATATTGTTATAA
*URA3* Reporter Position (kb)^a^	Primer	Sequence
C-20	SS1-F	TATGACAAGTTGCTTCCTGTCTTTATGGTCTGAGAGCAGTAAAAAAGCTTCATGGTAACTTTATTAACATACCCTACAACCTTAGTAGTTGGT
	SS1-R	ATGCATGGCTTAAAGAAAATACATTGCTGAAGCAAAAGCTCACCAAAAATTTGCGTTGCAAAGCTTTGTTTGCAGTTTTGCTGGCCGCATCT
C-7.2	SS2-F	TCTCTAAACTATGATTTGGACACATTTACGGATAGATTACCTAACGCTGGAGAAACTTTAGTTTTGCTGGCCGCATCT
*URA3* Reporter Position (kb)^a^	Primer	Sequence
C-7.2	SS2-R	ATGGCCGCAGCTTGGTTCAATCCCTTACCACCAGCATGTGTTTCGAAGTGGTTAGCCCTGCTACAACCTTAGTAGTTG.
C-5.8	SS3-F	TTGTCACAGGTGTTTTGTTGGGTGTTAAAACTTTCAATGACCCTGTCGAACACCGGTGTATAGTTTTGCTGGCCGCATCT
	SS3-R	TGTGATGTGTAATGGAATGGCTTCACTAGCCCATAAGAAAGCACAGCATTCTACCAATGCCCTACAACCTTAGTAGTTGG
T-7.1	SS4-F	TGCATTGTTATTCCTTCGAAACCAGCAGTAATAAGTCGTCCTGAGAACGATGTAGATTTACTACAACCTTAGTAGTTGGT
	SS4-R	CAAGAAGAAGGACGACTTTAAGATGGAAGGAGGTGATTTAGAGTACCAAC ATGTAAAGATCAGTTTTGCTGGCCGCATCT
T-9.1	SS5-F	AAAAACTAATGTTGACATGACATTATGCAATAATTTATTTATGAGAGAAAGAGAAAAACACTACAACCTTAGTAGTTGGT
	SS5-R	TGTCACACTACCTCTTGGTGAGATTCGATTGAAGAGGCGTGATTTTATGAAAAATTTGTAGAGTTTTGCTGGCCGCATCT
T-11.5	SS6-F	TATACACGCACTATTTTTCTTATATACAGGAGATGGGTGGCCACAGAACCCGCGCCTAGCCTACAACCTTAGTAGTTGGT
	SS6-R	TACATCAGAGGTACAAGGTTGGATGTCGGCGACCTCGAGGCAGGTCTAAGAAGAAAGAAATAGAGTTTTGCTGGCCGCATCT
T-14.5	SS7-F	CTTTTCGTTTGGTGTCTGTTCAGGGCCCAGTATCATTATGACGTTGTACCTGACCTTATGACTACAACCTTAGTAGTTGGT
	SS7-R	CCATTCCCTATTGTATATCTATCAAGGGCTTGCGAGGGACACACGTGGTATGGTGGCAGTAGTTTTGCTGGCCGCATCT
Sequencing Location (kb)^a^	Primers	Sequence
T-7.1	SEQ-1	CAAGAAGAAGGACGACTTTAAGATG
	SEQ-2	TGCATTGTTATTCCTTCGAAACCAGC
	SEQ-3	AGTCAAATTGCAGTACTCT
	SEQ-4	ATTCGTAATGTCTGCCCAT
C-5.8	SEQ-3	AGTCAAATTGCAGTACTCT
Sequencing Location (kb)^a^	Primers	Sequence
C-5.8	SEQ-4	ATTCGTAATGTCTGCCCAT
	SEQ-5	GGACAGTTAGAAGGGGAACCAATAAAC
	SEQ-6	AATTGAAAATTTGGCAGTTCCTAAGCTA

^a^ Depicts the position of the *URA3* reporter gene from the break site in kilobases

“T” represents the telomeric side of the HO-break site. “C” refers to the centromeric side of the HO-break site.

Additionally, we performed the reconstruction experiments to illustrate the efficiency and the reproducibility of our mutagenesis detection method that involves replica plating rare mutant cells to FOA medium after 12 h of growth on YEP-GAL medium. Briefly, yeast cells with a wild-type *URA3* gene and the mutated HO cleavage site at the *MAT* locus (URA+, FOA sensitive) were mixed with cells with mutations in *ura3* and the HO site (URA-, FOA resistant) at two different ratios (10^5^:1 and 10:1), plated onto YEP-GAL and FOA-GAL and grew them at 30^°^C for 12 h. The YEP-GAL plates were then replica-plated to FOA containing medium as described in our experimental protocol and incubated at 30^°^C for three more days. We scored the number of colonies on FOA plates and divided by the number of colonies grown on YEP-GAL plates. The median frequencies and the 95% confidence intervals were determined using the web tool “FALCOR”. We performed the experiments a total of three times to test the reproducibility of the mutagenesis measurement.

As shown in S24 Table, the mutation frequencies calculated by replica plating led to ~40% as compared to those analyzed by direct plating to FOA plates. The results suggest that the replica plating efficiency might correspond to approximately 50%. Most importantly, the mutation frequencies measured by the replica plating are remarkably constant and highly reproducible in three different tests with two different ratios of FOA+/- cell populations.

## Supporting information

S1 Fig The cleavage efficiency of HO-endonuclease. The cleavage efficiency was calculated by quantitative real time PCR using primers across the HO-endonuclease recognition site. Cells are harvested at different time points (0-, 2-, 4-, 6-, 8- and 10-h) after HO endonuclease induction. The X-axis represents time (hours) after galactose was added to the cells. The Y-axis represents the percentage of uncut DNA at each time point.
(PDF)

S2 Fig Mutation spectrum of spontaneous 5-FOA-resistant mutations at the *URA3* locus (7.1-kb from the HO-target site, SS4 strain). The antisense (unresected) strand of the 804-bp *URA3* open reading frame is shown. All mutations are generated under no DSB conditions. The sequence changes observed in independent *ura3* mutants are depicted above the sequence in green. Letters indicate single base substitutions, open triangles indicate single base deletions, and solid triangles indicate insertions.
(PDF)

S3 Fig Mutation spectrum of DNA double strand break-induced 5-FOA-resistant mutations at the *URA3* locus (7.1-kb from the HO-break site) in a strain lacking MH (SS1) across the break point junction. The antisense (unresected) strand of the 804-bp *URA3* open reading frame is shown as described in S2 Fig. All mutations are generated under DSB conditions. The sequence changes observed in independent *ura3* mutants are depicted above the sequence in orange. Letters indicate single base substitutions, open triangles indicate single base deletions, and short lines above the sequence indicate multiple base deletions (2–3 bp). Solid triangles indicate insertions.
(PDF)

S4 Fig Mutation spectrum of DNA double strand break-induced 5-FOA-resistant mutations at the *URA3* locus (7.1-kb from the HO-break site) in a strain with 15- (SS4) or 203-bp repeats (SS2) across the break point junction. The antisense (unresected) strand of the 804-bp *URA3* open reading frame is shown as described in S2 Fig. All mutations are generated under DSB conditions. The sequence changes observed in independent *ura3* mutants for MMEJ repair (15-bp MH) are depicted above the sequence in blue and below the sequence for SSA repair (203-bp repeat) in red. Letters indicate single base substitutions, open triangles indicate single base deletions, and short lines above the sequence indicate multiple base deletions (2–3 bp). Solid triangles indicate insertions.
(PDF)

S5 Fig Cell cycle analysis by flow cytometry. Cell cycle profiles at different time points for 15- (**A, D**), 18- (**B, E**) and 203-bp (**C, F**) homology strains, respectively. Cells were arrested in G2 by treatment with nocodazole (20 μg/ml) before (**D**-**F)** or after (**A-C**) HO expression. Cells were harvested at indicated time points and fixed in 70% ethanol. DNA was labeled with propidium iodide, and cellular DNA content was analyzed using a FACScalibur machine.
(PDF)

S6 Fig Repair kinetics. **A**. DSB repair kinetics detected by quantitative real time PCR in G2 arrested cells. Cells were arrested in G2 by treatment with nocodazole (20 μg/ml) in YEP-glycerol media for 2.5 h prior to HO endonuclease induction by 2% galactose. Cells were harvested at indicated time points (0, 2, 4, 6 and 8 h) after HO-endonuclease induction. The X-axis represents time (hours) after galactose was added to the cells. **B-C**. Relative DSB repair kinetics in G2 arrested cells. Relative DSB repair kinetics in G2 arrested cells by nocodazole treatment before (**B**) and after (**C**) HO expression. Relative repair kinetics was calculated by dividing the level of repair products at the indicated time by the amount of repair products at 8 h and 10 h post-HO expression.
(PDF)

S7 Fig Mutation spectrum of spontaneous 5-FOA-resistant mutations at the *URA3* locus (7.1-kb from the HO-target site, SS4) after 20 J/m2 UV treatment. The antisense (unresected) strand of the 804-bp *URA3* open reading frame is shown as described in S2 Fig. All mutations are generated under no DSB conditions. The sequence changes observed in independent *ura3* mutants are depicted above the sequence in green. Letters indicate single base substitutions, open triangles indicate single base deletions, and short lines above the sequence indicate multiple base deletions (2–3 bp). Solid triangles indicate insertions.
(PDF)

S8 Fig Mutation spectrum of DSB-induced 5-FOA-resistant mutations at the *URA3* locus (7.1-kb from the HO-break site) in a strain with 15- (SS4) or 203-bp repeats (SS2) across the break point junctions after 20 J/m2 UV treatment. The antisense (unresected) strand of the 804-bp *URA3* open reading frame is shown as described in S2 Fig. All mutations are generated under DSB conditions. The sequence changes observed in independent *ura3* mutants for MMEJ repair are depicted above the sequence in blue and below the sequence for SSA repair in red. Letters indicate single base substitutions, open triangles indicate single base deletions, and short lines above or below the sequence indicate multiple base deletions (2–3 bp). Solid triangles indicate insertions.
(PDF)

S9 Fig MMEJ induces chromosomal translocations and mutagenesis. **A**. Graph illustrating the survival frequency of yeast strains upon persistent HO expression that induced DSBs at two different chromosomes, chromosome III and V. The types of repair events were determined based on hygromycin sensitivity; hygromycin-sensitive (MMEJ) and resistant (NHEJ). Survival frequency was calculated by dividing the number of colonies on galactose containing plates by the number of colonies plated onto YEP-dextrose. The graph also demonstrates the fraction of intra- and inter-chromosomal repair events. The results are the average of three independent experiments. 100 colonies from each survival experiment were assessed by PCR to detect intra- or inter-chromosomal repair products. **B**. Types of FOA^R^ survivors after persistent HO expression. The percentage of intra- vs inter-chromosomal repair events and the status of the hygromycin gene are plotted. To induce a persistent DSB, cells were plated onto YEP-galactose and subsequently replica plated onto 5-Fluoroorotic Acid (5-FOA) plates. 100 colonies from each experiment were analyzed by PCR to detect intra- or inter-chromosomal repair products. The results are the average of three independent experiments. **C.** The percentage of intra- vs inter-chromosomal repair events upon 2 h HO expression among hph- trp- survivors. The results are the average of three independent experiments. 100 colonies from each survival experiment were assessed by PCR to detect intra- or inter-chromosomal repair products.
(PDF)

S10 Fig Mutation spectrum of 5-FOA-resistant mutations at the *URA3* locus (7.1-kb from the HO-break site, SS17INTER7.1) on chromosome III following two simultaneous HO breaks induced flanking 17 bp MH across the break point junction. The antisense (unresected) strand of the 804-bp *URA3* open reading frame is shown. All mutations are generated under DSB conditions. The sequence changes observed in independent *ura3* mutants for HYG^R^ events are depicted above the sequence in blue and below the sequence for HYG^S^ in red. Letters indicate single base substitutions, open triangles indicate single base deletions, and short lines above the sequence indicate multiple base deletions (2–4 bp). Solid triangles indicate insertions.
(PDF)

S11 Fig Model for MMEJ-mediated mutagenesis. Upon DNA break induction, end resection reveals MHs flanking the break site, and leads to annealing via MHs. Due to the instability of strand annealing via short MHs, repair is delayed and resection persists until it forms extensive single stranded DNA that is vulnerable to DNA damage and mutagenesis if DNA synthesis across the lesions ensues by a translesion polymerase.
(PDF)

S1 Table Percentage survival of yeast mutants. ^**a**^ Depicts the position of the *URA3* reporter gene from the break site in kilobases. “T” represents telomeric side of the HO-break site. “C” refers to centromeric side of the HO-break site.
^**b**^ Depicts the size of homology flanking the HO-cleavage site.
^**c**^ Percentage of survival was calculated as described in Materials and Methods from the average of three independent experiments. SD, Standard deviation.
(PDF)

S2 Table Median frequencies of *ura3* mutants (FOA^R^) and 95% Confidence Interval (95% CI) were calculated by Fluctuation Analysis Calculator (FALCOR). ^**a**^ Depicts the position of the *URA3* reporter gene from the break site in kilobases. “T” represents telomeric side of the HO-break site. “C” refers to centromeric side of the HO-break site.
^**b**^ Depicts the size of homology flanking the HO-cleavage site.
^**c**^ GLU refers to glucose containing media. HO-endonuclease not expressed, thus representing no-break conditions.
^**d**^ GAL refers to 2% galactose containing media. Galactose induces the expression of HO-endonuclease, thus generating double strand breaks (DSBs).
^**e**^ Fold represents the increase in mutation frequency after “GAL” over “GLU” control. The numbers in parentheses indicate the mutation frequency relative to that in the no-homology strain.
*Strain with no HO cut site.
^f^ 2 h induction of HO-endonuclease in 2% galactose containing media.
NA-Not Available-Persistent HO-endonuclease induction leads to no viable FOA^R^ colonies.
(PDF)

S3 Table Median frequencies of *can1* mutants (CAN^R^) and 95% Confidence Interval (95% CI) were calculated by Fluctuation Analysis Calculator (FALCOR). ^**a**^ Depicts the position of the *URA3* reporter gene from the break site in kilobases. “T” represents telomeric side of the HO-break site. “C” refers to centromeric side of the HO-break site.
^**b**^ Depicts the size of homology flanking the HO-cleavage site.
^**c**^ GLU refers to glucose containing media. HO-endonuclease not expressed, thus representing no-break conditions.
^**d**^ GAL refers to 2% galactose containing media. Galactose induces the expression of HO-endonuclease, thus generating double strand breaks (DSBs).
^**e**^ Fold represents the increase in mutation frequency “GAL” over “GLU” control. The numbers in parentheses indicate the mutation frequency relative to that in the no-homology strain.
*Strain with no HO cut site.
^f^ 2 h induction of HO-endonuclease in 2% galactose containing media.
(PDF)

S4 Table Analysis of *ura3* mutation events from FOA^R^ survivors upon HO expression. The reporter is located at the **7.1 kb** telomere-proximal location.
^a^ Mutations were identified by sequencing of repair events from FOA^R^ colonies.
^**b**^ GLU refers to glucose containing media.
^**c**^ GAL refers to galactose containing media.
bp, base pairs; Pyr:Pur, ratio between Pyrimidine vs Purine mutations; In-Del, insertions and deletions.
(PDF)

S5 Table Analysis of *ura3* mutation events from FOA^R^ survivors upon HO expression. The reporter is located at the **5.8 kb** centromere-proximal location.
^a^ Mutations were identified by sequencing of repair events from FOA^R^ colonies.
^**b**^ GLU refers to glucose containing media.
^**c**^ GAL refers to galactose containing media.
bp, base pairs; Pyr:Pur, ratio between Pyrimidine vs Purine mutations; In-Del, insertions and deletions.
(PDF)

S6 Table Median frequencies of *ura3* mutants (FOA^R^) and 95% Confidence Interval (95% CI) with 20 J/m2 UV treatment were calculated by Fluctuation Analysis Calculator (FALCOR). ^**a**^ Depicts the position of the *URA3* reporter gene from the break site in kilobases. “T” represents telomeric side of the HO-break site. “C” refers to centromeric side of the HO-break site.
^**b**^ Depicts the size of homology flanking the HO-cleavage site.
^**c**^ GLU refers to glucose containing media. HO-endonuclease not expressed, thus representing no-break conditions.
^**d**^ GAL refers to 2% galactose containing media. Galactose induces the expression of HO-endonuclease, thus generating breaks.
^**e**^ Fold represents the increase in mutation frequency after “GAL” over “GLU” control. The numbers in parentheses indicate the mutation frequency relative to that in the no-homology strain.
^f^ 2 h induction of HO-endonuclease in 2% galactose containing media.
(PDF)

S7 Table Median frequencies of *can1* mutants (CAN^R^) and 95% Confidence Interval (95% CI) with 20 J/m2 UV treatment were calculated by Fluctuation Analysis Calculator (FALCOR). ^**a**^ Depicts the position of the *URA3* reporter gene from the break site in kilobases. “T” represents telomeric side of the HO-break site. “C” refers to centromeric side of the HO-break site.
^**b**^ Depicts the size of homology flanking the HO-cleavage site.
^**c**^ GLU refers to glucose containing media. HO-endonuclease not expressed, thus representing no-break conditions.
^**d**^ GAL refers to 2% galactose containing media. Galactose induces the expression of HO-endonuclease, thus generating double strand breaks (DSBs).
^**e**^ Fold represents the increase in mutation frequency “GAL” over “GLU” control. The numbers in parentheses indicate the mutation frequency relative to that in the no-homology strain.
^f^ 2 h induction of HO-endonuclease in 2% galactose containing media.
(PDF)

S8 Table Analysis of *ura3* mutation events from FOA^R^ survivors upon HO expression and UV irradiation. The reporter is located at the **7.1 kb** telomere-proximal location.
^a^ Mutations identified by sequencing of FOA^R^ colonies with 20 J/m^2^ UV treatment.
^**b**^ GLU refers to glucose containing media.
^**c**^ GAL refers to galactose containing media.
bp, base pairs; Pyr:Pur, ratio between Pyrimidine vs Purine mutations; In-Del, insertions and deletions.
(PDF)

S9 Table Analysis of *ura3* mutation events from FOA^R^ survivors upon two simultaneous HO cleavage events. The reporter is located at the **7.1 kb** telomere-proximal location.
^a^ Mutations identified by sequencing of FOA^R^ events in a strain carrying two HO cleavage sites on two different chromosomes after HO expression.
^**b**^ GAL refers to galactose containing media.
bp, base pairs; HYG^R,^ hygromycin resistant; HYG^S^, hygromycin sensitive; Pyr:Pur, ratio between Pyrimidine vs Purine mutations; In-Del, insertions and deletions.
(PDF)

S10 Table List of strains used in the study. (PDF)

S11 Table Mutation spectra in the category of FOA^R^ mutants. The mutations were tabulated from the FOA^R^ colonies in the glucose containing media (GLU) with the 15 bp MH at telomeric 7.1 kb location.
(XLSX)

S12 Table Mutation spectra in the category of FOA^R^ mutants. The mutations were tabulated from the FOA^R^ colonies in the galactose containing media (GAL) with no MH at telomeric 7.1 kb location.
(XLSX)

S13 Table Mutation spectra in the category of FOA^R^ mutants. The mutations were tabulated from the FOA^R^ colonies in the galactose containing media (GAL) with the 15 bp MH at telomeric 7.1 kb location.
(XLSX)

S14 Table Mutation spectra in the category of FOA^R^ mutants. The mutations were tabulated from the FOA^R^ colonies in the galactose containing media (GAL) with the 203 bp repeat at telomeric 7.1 kb location.
(XLSX)

S15 Table Mutation spectra in the category of FOA^R^ mutants. The mutations were tabulated from the FOA^R^ colonies in the glucose containing media (GLU) with the 15 bp MH at centromeric 5.8 kb location.
(XLSX)

S16 Table Mutation spectra in the category of FOA^R^ mutants. The mutations were tabulated from the FOA^R^ colonies in the galactose containing media (GAL) with the 15 bp MH at centromeric 5.8 kb location.
(XLSX)

S17 Table Mutation spectra in the category of FOA^R^ mutants. The mutations were tabulated from the FOA^R^ colonies in the glucose containing media (GLU) with the 15 bp MH at telomeric 7.1 kb location upon 20 J/m^2^ UV.
(XLSX)

S18 Table Mutation spectra in the category of FOA^R^ mutants. The mutations were tabulated from the FOA^R^ colonies in the galactose containing media (GAL) with the 15 bp MH at telomeric 7.1 kb location upon 20 J/m^2^ UV.
(XLSX)

S19 Table Mutation spectra in the category of FOA^R^ mutants. The mutations were tabulated from the FOA^R^ colonies in the galactose containing media (GAL) with the 203 bp repeat at telomeric 7.1 kb location upon 20 J/m^2^ UV.
(XLSX)

S20 Table Mutation spectra in the category of FOA^R^ mutants. The mutations were tabulated from the FOA^R^ colonies in the glucose containing media (GLU) with the 15 bp MH at centromeric 5.8 kb location upon 20 J/m^2^ UV.
(XLSX)

S21 Table Mutation spectra in the category of FOA^R^ mutants. The mutations were tabulated from the FOA^R^ colonies in the galactose containing media (GAL) with the 15 bp MH at centromeric 5.8 kb location upon 20 J/m^2^ UV.
(XLSX)

S22 Table Mutation spectra in the category of FOA^R^ mutants. The mutations were tabulated from the FOA^R^ colonies in the galactose containing media (GAL) and hygromycin resistant (HYG^R^).
(XLSX)

S23 Table Mutation spectra in the category of FOA^R^ mutants. The mutations were tabulated from the FOA^R^ colonies in the galactose containing media (GAL) and hygromycin sensitive (HYG^S^).
(XLSX)

S24 Table Efficiency of replica-plating. ^a^ represents the ratio of yeast cells with a wild-type *URA3* gene and cells with mutations in *ura3* (both with mutated HO cleavage site at the *MAT* locus).
^b^ represents the average frequency of FOA^R^ cells upon direct plating on FOA-GAL.
^c^ represents the median FOA^R^ frequency and 95% Confidence Interval of cells plated on YEP-GAL followed by replica plating.
^d,e^ Median frequencies of *ura3* mutants (FOA^R^) and 95% Confidence Interval (95% CI) were calculated by Fluctuation Analysis Calculator (FALCOR).
(PDF)
