# Supplementary figures and images for: Microhomology-mediated end joining induces hypermutagenesis at breakpoint junctions
Source: PLoS Genet. 2017 Apr 18;13(4):e1006714. doi: 10.1371/journal.pgen.1006714 (PMC5413072; doi:10.1371/journal.pgen.1006714)

**Figure S1**

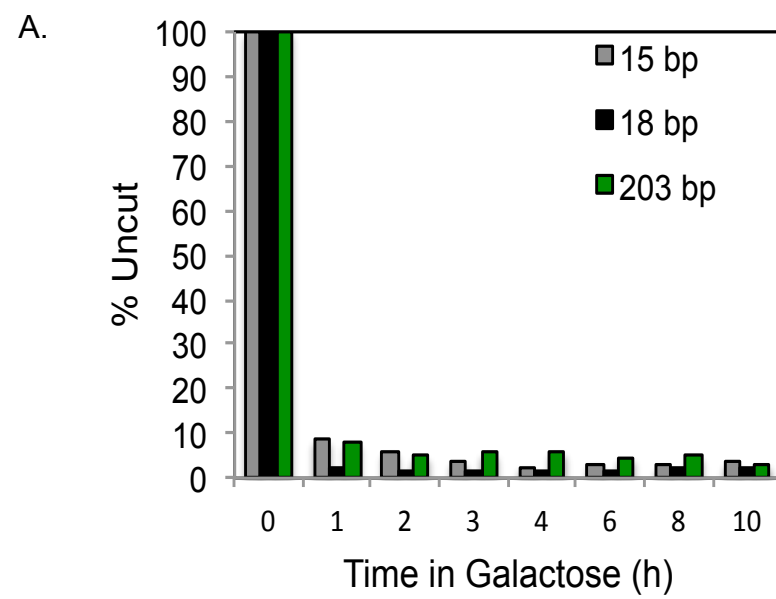

Supplement: S1 Fig — The cleavage efficiency was calculated by quantitative real time PCR using primers across the HO-endonuclease recognition site. Cells are harvested at different time points (0-, 2-, 4-, 6-, 8- and 10-h) after HO endonuclease induction. The X-axis represents time (hours) after galactose was added to the cells. The Y-axis represents the percentage of uncut DNA at each time point. (PDF) [file pgen.1006714.s001.pdf]

Figure S4

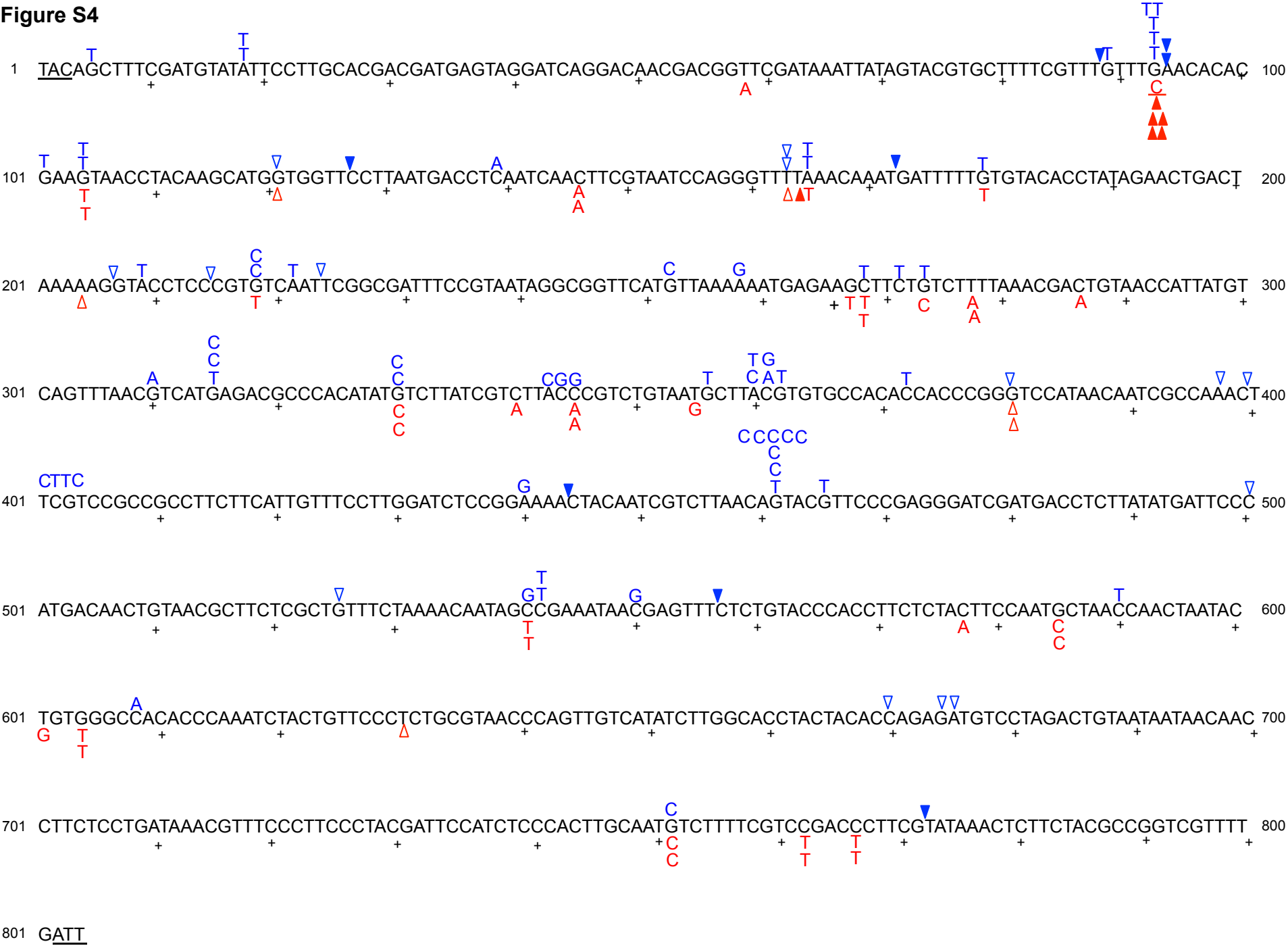

Supplement: S4 Fig — The antisense (unresected) strand of the 804-bp URA3 open reading frame is shown as described in S2 Fig. All mutations are generated under DSB conditions. The sequence changes observed in independent ura3 mutants for MMEJ repair (15-bp MH) are depicted above the sequence in blue and below the sequence for SSA repair (203-bp repeat) in red. Letters indicate single base substitutions, open triangles indicate single base deletions, and short lines above the sequence indicate multiple base deletions (2–3 bp). Solid triangles indicate insertions. (PDF) [file pgen.1006714.s004.pdf]

Figure S5

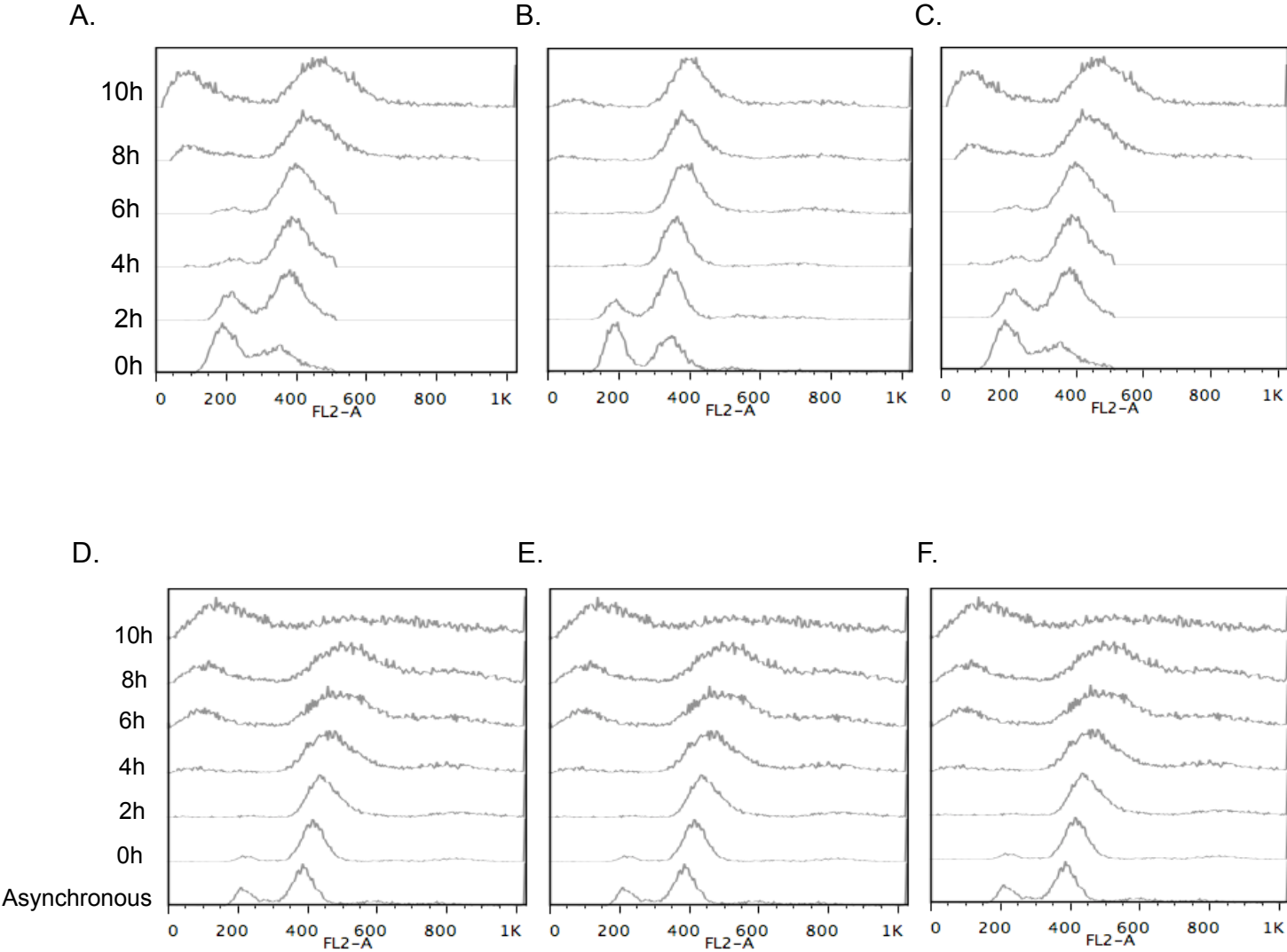

Supplement: S5 Fig — Cell cycle profiles at different time points for 15- (A, D), 18- (B, E) and 203-bp (C, F) homology strains, respectively. Cells were arrested in G2 by treatment with nocodazole (20 μg/ml) before (D-F) or after (A-C) HO expression. Cells were harvested at indicated time points and fixed in 70% ethanol. DNA was labeled with propidium iodide, and cellular DNA content was analyzed using a FACScalibur machine. (PDF) [file pgen.1006714.s005.pdf]

Figure S6

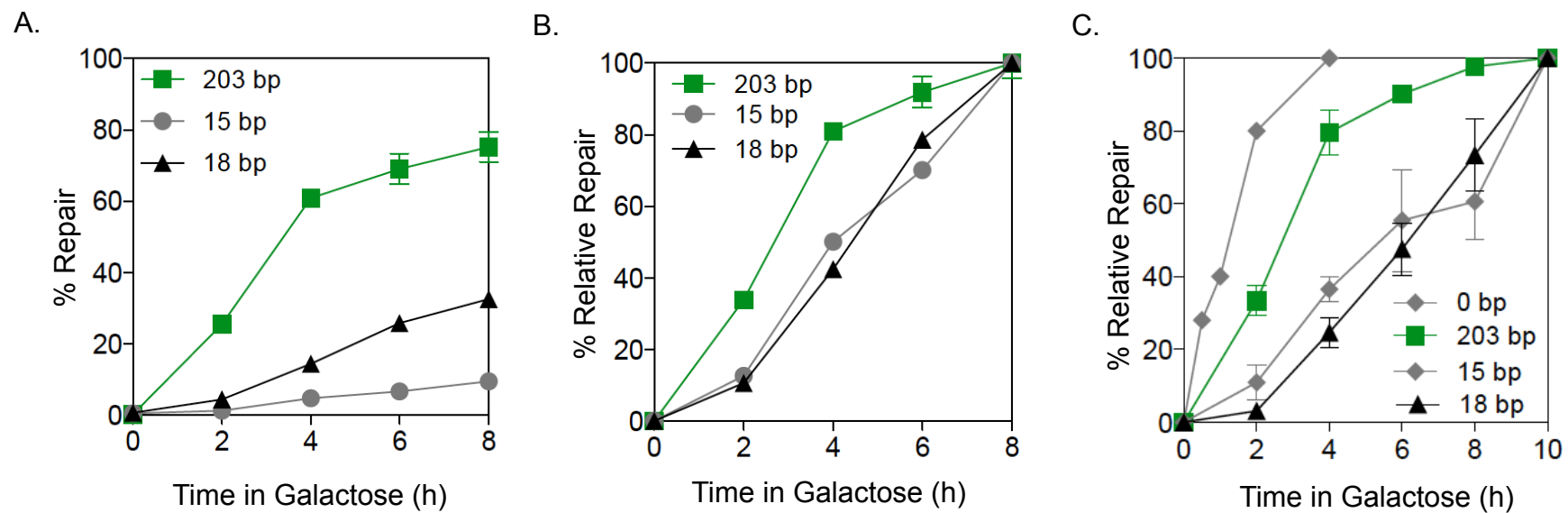

Supplement: S6 Fig — A. DSB repair kinetics detected by quantitative real time PCR in G2 arrested cells. Cells were arrested in G2 by treatment with nocodazole (20 μg/ml) in YEP-glycerol media for 2.5 h prior to HO endonuclease induction by 2% galactose. Cells were harvested at indicated time points (0, 2, 4, 6 and 8 h) after HO-endonuclease induction. The X-axis represents time (hours) after galactose was added to the cells. B-C. Relative DSB repair kinetics in G2 arrested cells. Relative DSB repair kinetics in G2 arrested cells by nocodazole treatment before (B) and after (C) HO expression. Relative repair kinetics was calculated by dividing the level of repair products at the indicated time by the amount of repair products at 8 h and 10 h post-HO expression. (PDF) [file pgen.1006714.s006.pdf]

Figure S8

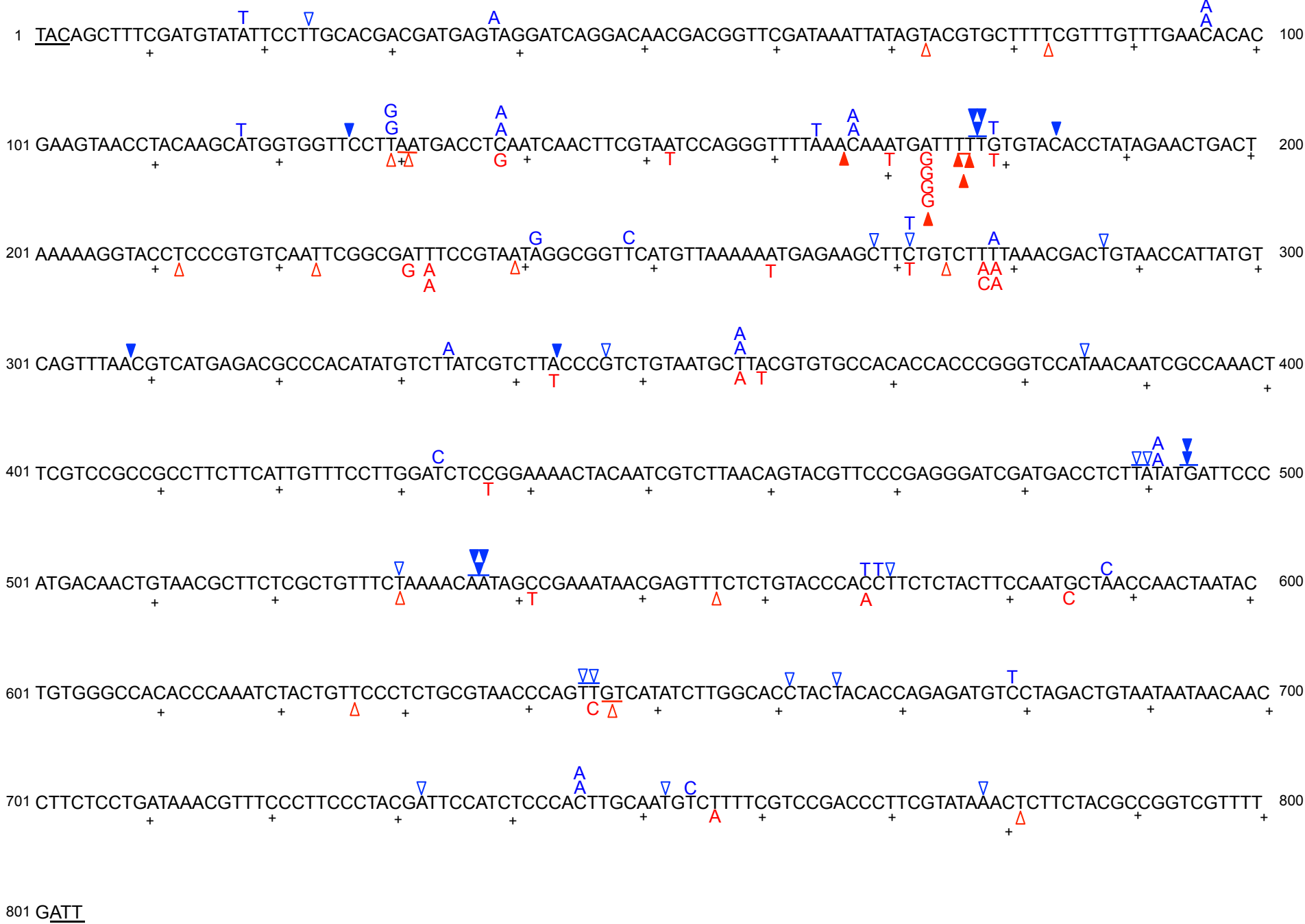

Supplement: S8 Fig — The antisense (unresected) strand of the 804-bp URA3 open reading frame is shown as described in S2 Fig. All mutations are generated under DSB conditions. The sequence changes observed in independent ura3 mutants for MMEJ repair are depicted above the sequence in blue and below the sequence for SSA repair in red. Letters indicate single base substitutions, open triangles indicate single base deletions, and short lines above or below the sequence indicate multiple base deletions (2–3 bp). Solid triangles indicate insertions. (PDF) [file pgen.1006714.s008.pdf]

Figure S9

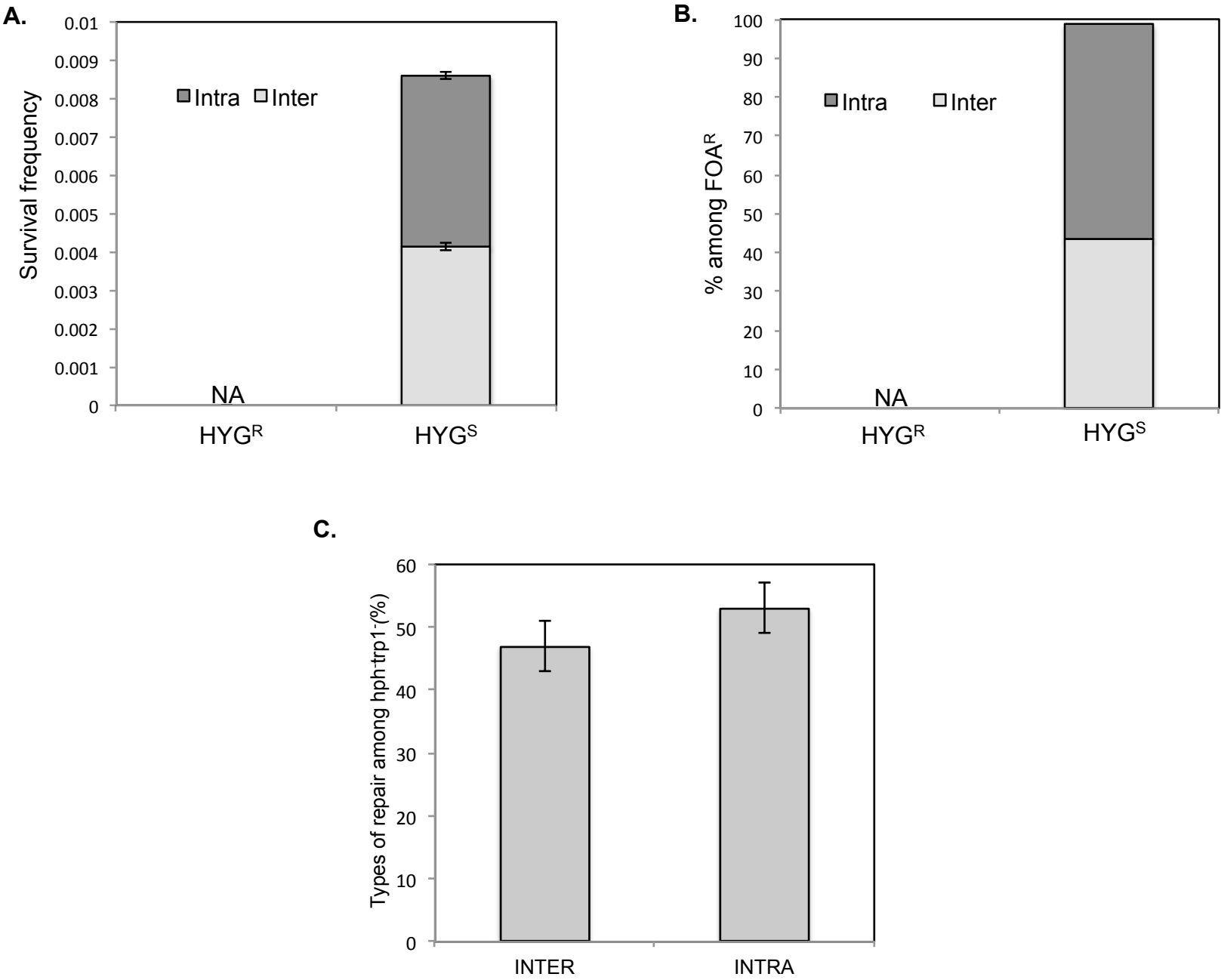

Supplement: S9 Fig — A. Graph illustrating the survival frequency of yeast strains upon persistent HO expression that induced DSBs at two different chromosomes, chromosome III and V. The types of repair events were determined based on hygromycin sensitivity; hygromycin-sensitive (MMEJ) and resistant (NHEJ). Survival frequency was calculated by dividing the number of colonies on galactose containing plates by the number of colonies plated onto YEP-dextrose. The graph also demonstrates the fraction of intra- and inter-chromosomal repair events. The results are the average of three independent experiments. 100 colonies from each survival experiment were assessed by PCR to detect intra- or inter-chromosomal repair products. B. Types of FOAR survivors after persistent HO expression. The percentage of intra- vs inter-chromosomal repair events and the status of the hygromycin gene are plotted. To induce a persistent DSB, cells were plated onto YEP-galactose and subsequently replica plated onto 5-Fluoroorotic Acid (5-FOA) plates. 100 colonies from each experiment were analyzed by PCR to detect intra- or inter-chromosomal repair products. The results are the average of three independent experiments. C. The percentage of intra- vs inter-chromosomal repair events upon 2 h HO expression among hph- trp- survivors. The results are the average of three independent experiments. 100 colonies from each survival experiment were assessed by PCR to detect intra- or inter-chromosomal repair products. (PDF) [file pgen.1006714.s009.pdf]

Figure S10

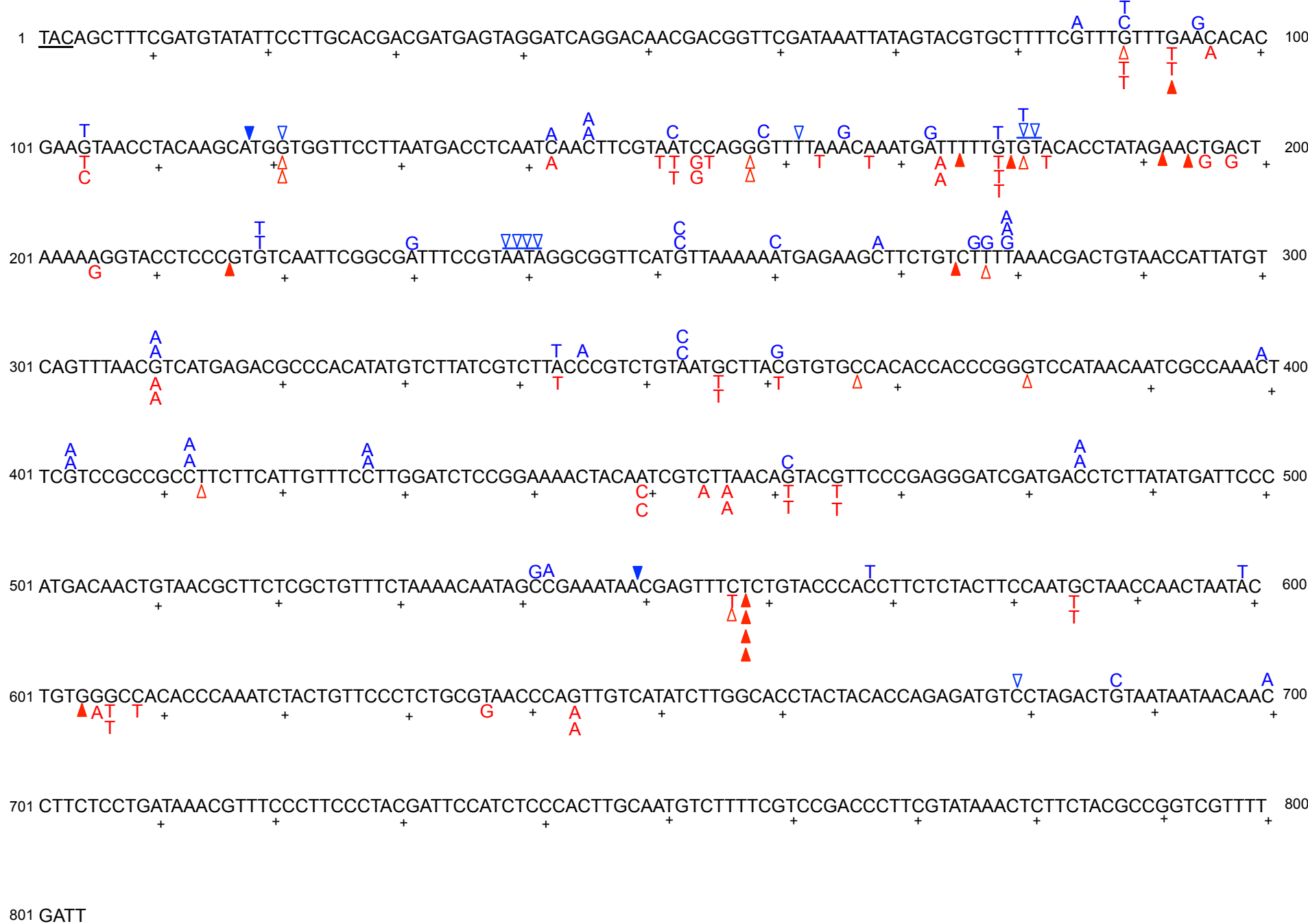

Supplement: S10 Fig — The antisense (unresected) strand of the 804-bp URA3 open reading frame is shown. All mutations are generated under DSB conditions. The sequence changes observed in independent ura3 mutants for HYGR events are depicted above the sequence in blue and below the sequence for HYGS in red. Letters indicate single base substitutions, open triangles indicate single base deletions, and short lines above the sequence indicate multiple base deletions (2–4 bp). Solid triangles indicate insertions. (PDF) [file pgen.1006714.s010.pdf]

Figure-S11

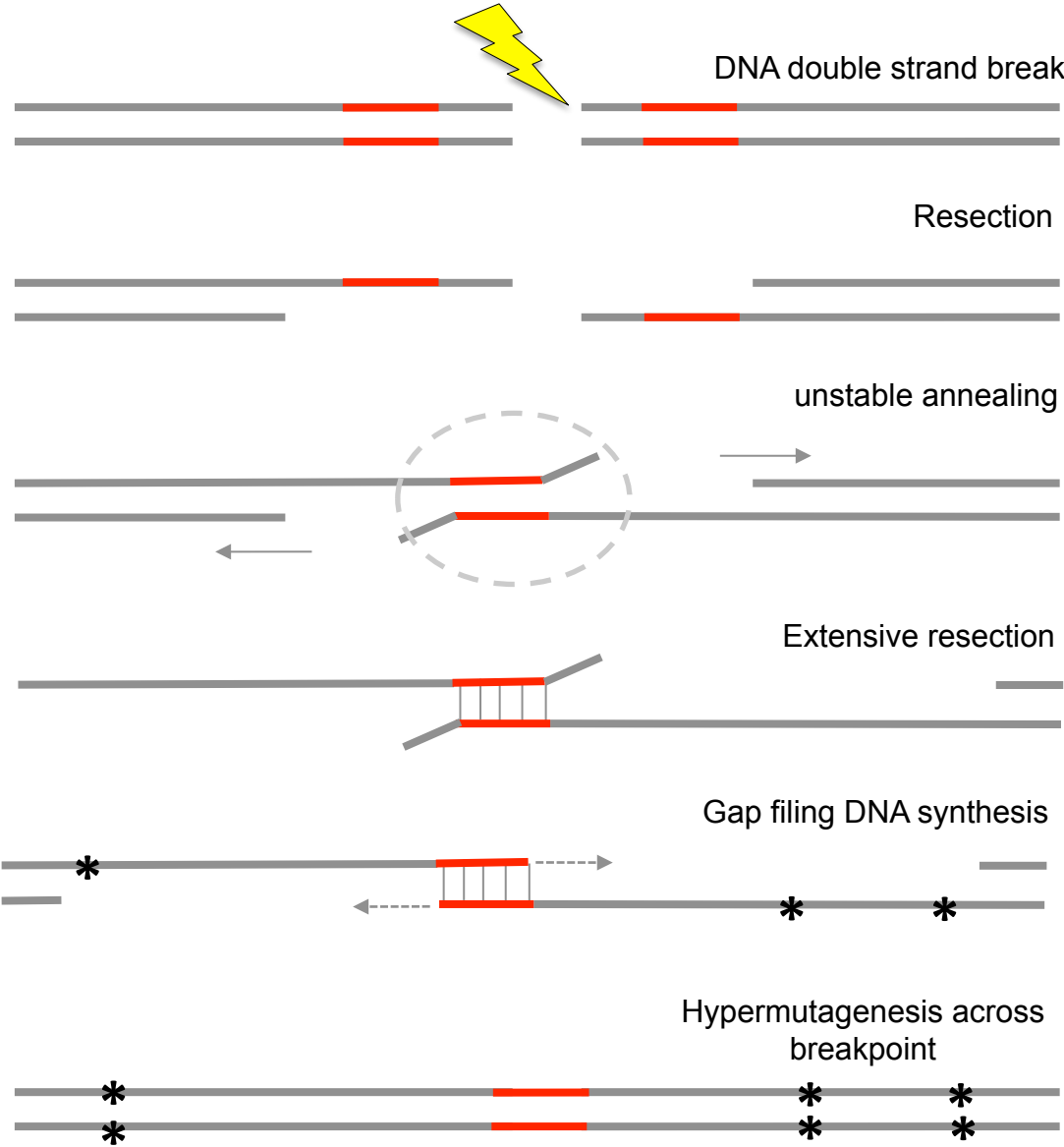

Supplement: S11 Fig — Upon DNA break induction, end resection reveals MHs flanking the break site, and leads to annealing via MHs. Due to the instability of strand annealing via short MHs, repair is delayed and resection persists until it forms extensive single stranded DNA that is vulnerable to DNA damage and mutagenesis if DNA synthesis across the lesions ensues by a translesion polymerase. (PDF) [file pgen.1006714.s011.pdf]
