# Supplementary material for: Microhomology-mediated end joining induces hypermutagenesis at breakpoint junctions
Source: PLoS Genet. 2017 Apr 18;13(4):e1006714. doi: 10.1371/journal.pgen.1006714 (PMC5413072; doi:10.1371/journal.pgen.1006714)
Supplement: S7 Fig — The antisense (unresected) strand of the 804-bp URA3 open reading frame is shown as described in S2 Fig. All mutations are generated under no DSB conditions. The sequence changes observed in independent ura3 mutants are depicted above the sequence in green. Letters indicate single base substitutions, open triangles indicate single base deletions, and short lines above the sequence indicate multiple base deletions (2–3 bp). Solid triangles indicate insertions. (PDF) [file pgen.1006714.s007.pdf]

Figure S7

1 TACAGCTTTCGATGTATATTCCTTGCACGACGATGAGTAGGATCAGGACAACGACGGTTCGATAAATTATAGTACGTGCTTTTCGTTTGTTTGAACACAC 100

101 GAAGTAACCTACAAGCATGGTGGTTCCTTAATGACCTCAATCAACTTCGTAATCCAGGGTTTTAAACAAATGATTTTTGTGTACACCTATAGAACTGACT 200

201 AAAAAGGTACCTCCCGTGTCAATTCGGCGATTTCCGTAATAGGCGGTTCATGTTAAAAAATGAGAAGCTTCTGTCTTTTAAACGACTGTAACCATTATGT 300

301 CAGTTTAACGTCATGAGACGCCCACATATGTCTTATCGTCTTACCCGTCTGTAATGCTTACGTGTGCCACACCACCCGGGTCCATAACAATCGCCAAACT 400

401 TCGTCCGCCGCCTTCTTCATTGTTTCCTTGGATCTCCGGAAAACTACAATCGTCTTAACAGTACGTTCCCGAGGGATCGATGACCTCTTATATGATTCCC 500

501 ATGACAACTGTAACGCTTCTCGCTGTTTCTAAAACAATAGCCGAAATAACGAGTTTCTCTGTACCCACCTTCTCTACTTCCAATGCTAACCAACTAATAC 600

601 TGTGGGCCACACCCAAATCTACTGTTCCCTCTGCGTAACCCAGTTGTCATATCTTGGCACCTACTACACCAGAGATGTCCTAGACTGTAATAATAACAAC 700

701 CTTCTCCTGATAAACGTTTCCCTTCCCTACGATTCCATCTCCCACTTGCAATGTCTTTTCGTCCGACCCTTCGTATAAACTCTTCTACGCCGGTCGTTTT 800

801 GATT
