# Supplementary material for: Microhomology-mediated end joining induces hypermutagenesis at breakpoint junctions
Source: PLoS Genet. 2017 Apr 18;13(4):e1006714. doi: 10.1371/journal.pgen.1006714 (PMC5413072; doi:10.1371/journal.pgen.1006714)
Supplement: S1 Table — a Depicts the position of the URA3 reporter gene from the break site in kilobases. “T” represents telomeric side of the HO-break site. “C” refers to centromeric side of the HO-break site. b Depicts the size of homology flanking the HO-cleavage site. c Percentage of survival was calculated as described in Materials and Methods from the average of three independent experiments. SD, Standard deviation. (PDF) [file pgen.1006714.s012.pdf]

Table S1 Percentage Survival of Yeast Mutants

Strain	Genotype	Position (kb)[a]	Size of Homology (bp)[b]	% Survival[c] Mean ± SD
MH15	Wild type	-	15	8.83 ± 2.20
SS4	Wild type	T-7.1	15	8.71 ± 2.90
SS5	Wild type	T-9.1	15	7.56 ± 1.36
SS6	Wild type	T-11.5	15	8.62 ± 0.89
SS7	Wild type	T-14.5	15	7.57 ± 0.76
SS8	Wild type	C-5.8	15	7.61 ± 0.71
SS9	Wild type	C-7.2	15	7.46 ± 1.39
SS10	Wild type	C-20	15	7.50 ± 0.91
SS11	*rev3Δ*	T-7.1	15	6.09 ± 0.61
SS12	*rev1Δ*	T-7.1	15	9.27 ± 1.58
SS13	*rad30Δ*	T-7.1	15	8.50 ± 1.24
SS14	*rev3Δrev1Δrad30Δ*	T-7.1	15	7.17 ± 1.35
SS15	*sgs1Δ*	T-7.1	15	10.15 ± 1.90
SS16	*exo1Δ*	T-7.1	15	9.90 ± 2.94
SS17	*pif1Δ*	T-7.1	15	23.80 ± 5.10
SS203	Wild type	-	203	81.90 ± 8.61
SS527	Wild type	-	527	71.90 ± 9.61
SS2	Wild type	T-7.1	203	82.70 ± 7.61
SS3	Wild type	T-11.5	203	91.19 ± 6.01
SS1	Wild type	T-7.1	0	0.18 ± 0.06
SS1-2	*lig4*Δ	T-7.1	0	0.009 ± 0.003
M18	Wild type	-	18	37.00 ± 4.10
M18-7	Wild type	T-7.1	18	38.00 ± 8.10

Strain	Genotype	Position (kb)[a]	Size of Homology (bp)[b]	% Survival[c] Mean ± SD
M18	Wild type	-	18	37.00 ± 4.10
18-14T15A	Wild type	-	16	1.16 ± 0.90
18-3A4A	Wild type	-	16	11.40 ± 2.00
18-3A15A	Wild type	-	16	3.91 ± 1.04
18-9G	Wild type	-	17	5.33 ± 1.21
18-10C11C	Wild type	-	16	4.01 ± 1.56
18-16T	Wild type	-	17	4.16 ± 2.12
18-9-T	Wild type	-	17	5.40 ± 1.39
18-3A	Wild type	-	17	18.20 ± 2.30
18-5G	Wild type	-	17	21.70 ± 2.80

[a] Depicts the position of the *URA3* reporter gene from the break site in kilobases. “T” represents telomeric side of the HO-break site. “C” refers to centromeric side of the HO-break site.

[b] Depicts the size of homology flanking the HO-cleavage site.

[c] Percentage of survival was calculated as described in Materials and Methods from the average of three independent experiments. SD, Standard deviation
