# Supplementary material for: Microhomology-mediated end joining induces hypermutagenesis at breakpoint junctions
Source: PLoS Genet. 2017 Apr 18;13(4):e1006714. doi: 10.1371/journal.pgen.1006714 (PMC5413072; doi:10.1371/journal.pgen.1006714)
Supplement: S2 Table — a Depicts the position of the URA3 reporter gene from the break site in kilobases. “T” represents telomeric side of the HO-break site. “C” refers to centromeric side of the HO-break site. b Depicts the size of homology flanking the HO-cleavage site. c GLU refers to glucose containing media. HO-endonuclease not expressed, thus representing no-break conditions. d GAL refers to 2% galactose containing media. Galactose induces the expression of HO-endonuclease, thus generating double strand breaks (DSBs). e Fold represents the increase in mutation frequency after “GAL” over “GLU” control. The numbers in parentheses indicate the mutation frequency relative to that in the no-homology strain. *Strain with no HO cut site. f 2 h induction of HO-endonuclease in 2% galactose containing media. NA-Not Available-Persistent HO-endonuclease induction leads to no viable FOAR colonies. (PDF) [file pgen.1006714.s013.pdf]

Table S2 Median frequencies of *ura3* mutants (FOA[R]) and 95% Confidence Interval (95% CI) were calculated by Fluctuation Analysis Calculator (FALCOR).

Strain	Genotype	Position (kb)[a]	Size of homology (bp)[b]	Frequency of FOA[R] mutants (X10^{-7})				
				GLU[c]		GAL[d]		Fold[e]
				MEDIAN	95% CI-range	MEDIAN	95% CI-range	
SS1		T-7.1	0	0.4	(0.3 - 0.6)	1.6	(1.2 - 2.7)[f]	4.1 (1.0)
SS1		T-7.1	0	1.0	(0.6 - 1.8)	<10	NA	NA
SS2		T-7.1	203	0.4	(0.2 - 1.5)	3.2	(2.1 - 5.2)	7.2 (1.8)
SS3		T-11.5	203	0.4	(0.1 - 1.0)	4.1	(2.8 - 8.7)	11.6 (2.8)
SS4		T-7.1	15	0.9	(0.2 - 2.9)	332.4	(273.8 - 453.3)	365.7 (88.7)
SS5		T-9.1	15	0.8	(0.3 - 1.6)	127.7	(52.6 - 235.3)	152.1 (36.9)
SS6		T-11.5	15	0.8	(0.4 - 1.9)	65.1	(57.3 - 95.5)	86.1 (20.9)
SS7		T-14.5	15	0.8	(0.1 - 2.0)	33.3	(16.9 - 48.6)	43.3 (10.5)
SS8		C-5.8	15	0.9	(0.2 - 2.9)	851.5	(788.9 - 1078.0)	931.0 (255.8)
SS9		C-7.2	15	0.3	(0.1 - 1.1)	87.8	(69.4 - 107.7)	331.1 (80.3)
SS10		C-20	15	6.8	(5.6 - 7.9)	34.5	(23.0 - 43.2)	5.1 (1.2)
SS11	*rev3Δ*	T-7.1	15	0.3	(0.1 - 0.4)	12.8	(4.5 - 31.5)	46.0 (11.2)
SS12	*rev1Δ*	T-7.1	15	0.2	(0.1 - 0.3)	25.7	(18.5 - 32.1)	150.5 (36.5)
SS13	*rad30Δ*	T-7.1	15	1.7	(0.7 - 2.4)	271.2	(226.7 - 325.8)	161.2 (39.1)
SS14	*rev3Δ rev1Δ rad30Δ*	T-7.1	15	1.0	(0.1 - 2.5)	43.6	(36.0 - 52.2)	43.3 (10.5)
SS15	*sgs1Δ*	T-7.1	15	1.9	(0.6 - 6.3)	25.0	(6.3 - 71.1)	13.1 (3.2)
SS16	*exo1Δ*	T-7.1	15	1.1	(0.3 - 2.2)	6.8	(2.3 - 12.0)	6.3 (1.5)
SS17	*pifΔ*	T-7.1	15	7.3	(4.2 - 8.0)	665.7	(281.7 - 852.3)	91.1 (22.1)
M18-7		T-7.1	18	1.8	(0.6 - 3.4)	24.9	(21.6 - 46.0)	13.5 (3.2)
SS18*		T-7.1		0.9	(0.6 - 1.3)	0.7	(0.2 - 1.5)	0.7 (0.1)

[a] Depicts the position of the *URA3* reporter gene from the break site in kilobases. “T” represents telomeric side of the HO-break site. “C” refers to centromeric side of the HO-break site.

[b] Depicts the size of homology flanking the HO-cleavage site.

[c] GLU refers to glucose containing media. HO-endonuclease not expressed, thus representing no-break conditions.

[d] GAL refers to 2% galactose containing media. Galactose induces the expression of HO-endonuclease, thus generating double strand breaks (DSBs)

[e] Fold represents the increase in mutation frequency after “GAL” over “GLU” control. The numbers in parentheses indicate the mutation frequency relative to that in the no-homology strain.

*Strain with no-HO cut site.

[f] 2h induction of HO-endonuclease in 2% galactose containing media and plated on YEPD.

NA-Not Available-Persistent HO-endonuclease induction leads to lack of FOA^R colonies.
