# Supplementary material for: Microhomology-mediated end joining induces hypermutagenesis at breakpoint junctions
Source: PLoS Genet. 2017 Apr 18;13(4):e1006714. doi: 10.1371/journal.pgen.1006714 (PMC5413072; doi:10.1371/journal.pgen.1006714)
Supplement: S3 Table — a Depicts the position of the URA3 reporter gene from the break site in kilobases. “T” represents telomeric side of the HO-break site. “C” refers to centromeric side of the HO-break site. b Depicts the size of homology flanking the HO-cleavage site. c GLU refers to glucose containing media. HO-endonuclease not expressed, thus representing no-break conditions. d GAL refers to 2% galactose containing media. Galactose induces the expression of HO-endonuclease, thus generating double strand breaks (DSBs). e Fold represents the increase in mutation frequency “GAL” over “GLU” control. The numbers in parentheses indicate the mutation frequency relative to that in the no-homology strain. *Strain with no HO cut site. f 2 h induction of HO-endonuclease in 2% galactose containing media. (PDF) [file pgen.1006714.s014.pdf]

Table S3 Median frequencies of *can1* mutants (CAN^R) and 95% Confidence Interval (95% CI) were calculated by Fluctuation Analysis Calculator (FALCOR).

Strain	Genotype	Position (kb)[a]	Size of homology (bp)[b]	Frequency of CAN^R mutants ($X10^{-7}$)				
				GLU[c]		GAL[d]		
				MEDIAN	95% CI-range	MEDIAN	95% CI-range	Fold[e]
SS1		T- 7.1	0	7.2	(5.0 - 9.5)	14.8	(9.9 - 21.7)[f]	2.1 (1.0)
SS1		T- 7.1	0	8.0	(4.8 - 9.3)	18.0	(10.5 - 24.6)	2.2 (1.1)
SS2		T-7.1	203	5.2	(4.7 - 9.1)	10.4	(6.8 - 11.3)	2.0 (1.0)
SS3		T-11.5	203	7.6	(4.7 - 9.5)	13.1	(10.0 - 17.8)	1.7 (0.8)
SS4		T-7.1	15	7.7	(3.7 - 13.5)	16.0	(8.4 - 35.8)	2.1 (1.0)
SS5		T-9.1	15	9.0	(6.8 - 19.4)	48.4	(33.7 - 64.7)	5.4 (2.6)
SS6		T-11.5	15	6.2	(5.5 - 17.2)	9.1	(7.1 - 23.2)	1.5 (0.7)
SS7		T-14.5	15	15.1	(11.1 - 20.8)	22.6	(11.6 - 29.5)	1.5 (0.7)
SS8		C-5.8	15	6.9	(5.1 - 10.5)	31.9	(14.1 - 81.6)	4.7 (2.2)
SS9		C-7.2	15	5.8	(3.1 - 14.8)	13.7	(5.4 - 19.0)	2.4 (1.1)
SS10		C-20	15	10.7	(9.2 - 17.6)	33.8	(25.5 - 47.4)	3.2 (1.5)
SS11	*rev3Δ*	T-7.1	15	4.5	(1.8 - 31.6)	10.6	(5.3 - 35.6)	2.4 (1.1)
SS12	*rev1Δ*	T-7.1	15	7.0	(3.6 - 18.9)	10.5	(8.2 - 24.4)	1.5 (0.7)
SS13	*rad30Δ*	T-7.1	15	12.4	(7.7 - 14.9)	27.0	(17.6 - 36.4)	2.2 (1.0)
SS14	*rev3Δ rev1Δ rad30Δ*	T-7.1	15	11.5	(6.6 - 26.7)	53.0	(37.6 - 72.8)	4.6 (2.2)
SS15	*sgs1Δ*	T-7.1	15	45.4	(30.7 - 77.6)	22.2	(9.4 - 50.0)	0.5 (0.2)
SS16	*exo1Δ*	T-7.1	15	69.0	(28.7 -137.4)	33.7	(9.8 - 64.9)	0.5 (0.2)
SS17	*pifΔ*	T-7.1	15	29.6	(8.1 - 49.0)	172.0	(148.0 - 261.4)	5.8 (2.8)
M18-7		T-7.1	18	15.7	(11.6 - 21.2)	14.5	(11.3 - 29.6)	0.9 (0.4)
SS18*		T-7.1		7.8	(4.2 - 14.5)	10.2	(7.0 - 11.4)	1.3 (0.6)

[a] Depicts the position of the *URA3* reporter gene from the break site in kilobases. “T” represents telomeric side of the HO-break site. “C” refers to centromeric side of the HO-break site.

[b] Depicts the size of homology flanking the HO-cleavage site.

[c] GLU refers to glucose containing media. HO-endonuclease not expressed, thus representing no-break conditions.

[d] GAL refers to 2% galactose containing media. Galactose induces the expression of HO-endonuclease, thus generating double strand breaks (DSBs).

[e] Fold represents the increase in mutation frequency after “GAL” over “GLU” control. The numbers in parentheses indicate the mutation frequency relative to that in the no-homology strain.

*Strain with no-HO cut site.

[f] 2h induction of HO-endonuclease in 2% galactose containing media and plated on YEPD.
