# Supplementary material for: Microhomology-mediated end joining induces hypermutagenesis at breakpoint junctions
Source: PLoS Genet. 2017 Apr 18;13(4):e1006714. doi: 10.1371/journal.pgen.1006714 (PMC5413072; doi:10.1371/journal.pgen.1006714)
Supplement: S4 Table — The reporter is located at the 7.1 kb telomere-proximal location. a Mutations were identified by sequencing of repair events from FOAR colonies. b GLU refers to glucose containing media. c GAL refers to galactose containing media. bp, base pairs; Pyr:Pur, ratio between Pyrimidine vs Purine mutations; In-Del, insertions and deletions. (PDF) [file pgen.1006714.s015.pdf]

Table S4 Analysis of *ura3* mutation events from FOA^R survivors upon HO expression. The reporter is located at the **7.1 kb** telomere proximal location

WT (Base in unresected strand)	Mutant base[a]	GLU[b]	GAL[c]		
		15bp	0bp	15bp	203 bp
A	G	1 (3.0%)	1 (2.3%)	2 (2.4%)	
A	T	3 (9.0%)	4 (9.3%)	7 (8.5%)	1 (2.2%)
A	C	1 (3.0%)	3 (6.9%)	2 (2.4%)	
Total A		5 (15.1%)	8 (18.6%)	11 (13.4%)	1 (2.2%)
G	A	3 (9.0%)		1 (1.2%)	
G	C		9 (20.9%)	14 (17.0%)	9 (20.0%)
G	T	2 (6.0%)	5 (11.6%)	17 (20.7%)	7 (15.5%)
Total G		5 (15.1%)	14 (32.5%)	32 (39.0%)	16 (35.5%)
T	C	1 (3.0%)		2 (2.4%)	
T	A	2 (6.0%)	1 (2.3%)		3 (6.6%)
T	G				2 (4.4%)
Total T		3 (9.0%)	1 (2.3%)	2 (2.4%)	5 (11.1%)
C	T	8 (24.2%)	1 (2.3%)	7 (8.5%)	8 (17.8%)
C	G		2 (4.6%)	5 (6.0%)	
C	A	7 (21.2%)	11 (25.5%)	3 (3.6%)	7 (15.6%)
Total C		15 (45.4%)	14 (32.5%)	15 (18.2%)	15 (33.3%)
Transition		13 (39.3%)	2 (4.6%)	12 (14.6%)	8 (17.7%)
Transversion		15 (45.4%)	35 (81.3%)	48 (58.5%)	29 (64.4%)
In/Del		5 (15.1%)	6 (13.9%)	22 (26.8%)	8 (17.7%)
Complex Mutations		NA	NA	NA	NA
Total Mutations		33	43	82	45
Total Sequenced		33	42	82	45
Pyr:Pur		18:10	15:22	17:43	20:17

[a] Mutations were identified by sequencing of repair events from FOA^R colonies.
[b] GLU refers to glucose containing media.
[c] GAL refers to galactose containing media.
NA Not Available- No events found

bp, base pairs; Pyr:Pur , ratio between Pyrimidine vs Purine mutations; In-Del, insertions and deletions
