# Supplementary material for: Microhomology-mediated end joining induces hypermutagenesis at breakpoint junctions
Source: PLoS Genet. 2017 Apr 18;13(4):e1006714. doi: 10.1371/journal.pgen.1006714 (PMC5413072; doi:10.1371/journal.pgen.1006714)
Supplement: S5 Table — The reporter is located at the 5.8 kb centromere-proximal location. a Mutations were identified by sequencing of repair events from FOAR colonies. b GLU refers to glucose containing media. c GAL refers to galactose containing media. bp, base pairs; Pyr:Pur, ratio between Pyrimidine vs Purine mutations; In-Del, insertions and deletions. (PDF) [file pgen.1006714.s016.pdf]

Table S5 Analysis of *ura3* mutation events from FOA[R] survivors upon HO expression. The reporter is located at the **5.8 kb** centromere proximal location

WT (Base in unresected strand)	Mutant base[a]	No U.V		20 J/m²U.V	
		GLU[b]	GAL[c]	GLU[b]	GAL[c]
		15bp	15bp	15bp	15 bp
A	G	1 (2.3%)			
A	T	1 (2.3%)	1 (2.2%)	1 (2.5%)	1 (2.2%)
A	C	2 (4.6%)	1 (2.2%)	2 (5.0%)	
Total A		4 (9.3%)	2 (4.5%)	3 (7.5%)	1 (2.2%)
G	A	7 (16.2%)	2 (4.5%)	11 (27.5%)	
G	C	2 (4.6%)	4 (9.0%)	6 (15.0%)	
G	T	12 (27.9%)	10 (22.7%)	9 (22.5%)	1 (2.2%)
Total G		21 (48.8%)	16 (36.3%)	26 (65.0%)	1 (2.2%)
T	C	1 (2.3%)		1 (2.5%)	12 (26.6%)
T	A	3 (6.9%)			12 (26.6%)
T	G	3 (6.9%)	2 (4.5%)	2 (5.0%)	2 (4.4%)
Total T		7 (16.2%)	2 (4.5%)	3 (7.5%)	26 (57.7%)
C	T	2 (4.6%)	2 (4.5%)	1 (2.5%)	2 (4.4%)
C	G	1 (2.3%)	2 (4.5%)	1 (2.5%)	
C	A	3 (6.9%)	1 (2.2%)	3 (7.5%)	3 (6.6%)
Total C		6 (13.9%)	5 (11.3%)	5 (12.5%)	5 (11.1%)
Transition		11 (25.5%)	4 (9.0%)	13 (32.5%)	14 (31.1%)
Transversion		27 (65.1%)	21 (47.7%)	24 (60.0%)	19 (46.6%)
In/Del		4 (9.3%)	14 (31.8%)	NA	9 (20.0%)
Complex mutations		1 (2.3%)	5 (11.3%)	3 (7.5%)	3 (6.6%)
Total Mutations		43	44	40	45
Total Sequenced		42	44	40	45
Pyr:Pur		13:25	7:18	8:29	31:2

[a] Mutations were identified by sequencing of repair events from FOA^R colonies.
[b] GLU refers to glucose containing media.
[c] GAL refers to galactose containing media.
NA Not Available- No events found

bp, base pairs; Pyr:Pur , ratio between Pyrimidine vs Purine mutations; In-Del, insertions and deletions
