# Supplementary material for: Microhomology-mediated end joining induces hypermutagenesis at breakpoint junctions
Source: PLoS Genet. 2017 Apr 18;13(4):e1006714. doi: 10.1371/journal.pgen.1006714 (PMC5413072; doi:10.1371/journal.pgen.1006714)
Supplement: S6 Table — a Depicts the position of the URA3 reporter gene from the break site in kilobases. “T” represents telomeric side of the HO-break site. “C” refers to centromeric side of the HO-break site. b Depicts the size of homology flanking the HO-cleavage site. c GLU refers to glucose containing media. HO-endonuclease not expressed, thus representing no-break conditions. d GAL refers to 2% galactose containing media. Galactose induces the expression of HO-endonuclease, thus generating breaks. e Fold represents the increase in mutation frequency after “GAL” over “GLU” control. The numbers in parentheses indicate the mutation frequency relative to that in the no-homology strain. f 2 h induction of HO-endonuclease in 2% galactose containing media. (PDF) [file pgen.1006714.s017.pdf]

Table S6 Median frequencies of *ura3* mutants (FOA^R) and 95% Confidence Interval (95% CI) with 20 J/m^2 U.V treatment were calculated by Fluctuation Analysis Calculator (FALCOR).

Strain	Genotype	Position (kb)[a]	Size of homology (bp)[b]	Frequency of FOA^R mutants (X10^{-7}) 20J U.V				
				GLU[c]		GAL[d]		
				MEDIAN	95% CI-range	MEDIAN	95% CI-range	Fold[e]
SS1		T-7.1	0	0.4	(0.3 - 0.6)	70.4	(26.9 - 183.5)[f]	170.8 (1.0)
SS1		T-7.1	0	1.0	(0.5 - 1.2)	22.9	(8.7 - 32.5)	22.9 (0.1)
SS2		T-7.1	203	0.5	(0.1 - 0.9)	139.0	(92.2 - 190.4)	268.1 (1.6)
SS3		T-11.5	203	0.2	(0.1 - 0.9)	62.6	(33.9 - 100.0)	276.0 (1.6)
SS4		T-7.1	15	0.4	(0.2 - 0.8)	35294.1	(24561.4 - 40344.8)	83814.1 (490.7)
SS5		T-9.1	15	0.7	(0.3 - 1.8)	20384.6	(16710.5 - 27500.0)	28893.9 (169.2)
SS6		T-11.5	15	0.6	(0.2 - 1.7)	1789.8	(1388.9 - 2296.8)	2804.0 (16.4)
SS7		T-14.5	15	0.7	(0.2 - 1.2)	199.4	(93.3 - 411.9)	279.1 (1.6)
SS8		C-5.8	15	1.3	(0.3 - 3.6)	27879.6	(22872.3 - 83681.2)	21815.0 (127.7)
SS9		C-7.2	15	0.8	(0.2 - 17.9)	12584.6	(6743.4 - 17015.4)	15855.6 (92.8)
SS10		C-20	15	6.9	(5.0 - 9.5)	328.5	(280.3 - 422.2)	47.5 (0.3)
SS11	*rev3Δ*	T-7.1	15	0.6	(0.4 - 0.9)	180.5	(33.4 - 222.6)	303.2 (1.8)
SS12	*rev1Δ*	T-7.1	15	0.6	(0.4 - 1.0)	126.7	(60.3 - 146.2)	215.3 (1.3)
SS13	*rad30Δ*	T-7.1	15	1.3	(0.6 - 3.0)	3160.0	(2929.8 - 4703.2)	2433.2 (14.2)
SS14	*rev3Δ rev1Δ rad30Δ*	T-7.1	15	0.8	(0.3 - 2.2)	93.5	(70.8 - 147.4)	112.2 (0.7)
SS15	*sgs1Δ*	T-7.1	15	4.8	(1.9 - 13.3)	108.5	(37.3 - 196.4)	22.6 (0.1)
SS16	*exo1Δ*	T-7.1	15	1.0	(0.3 - 3.5)	11.1	(8.1 - 14.2)	11.0 (0.1)
SS17	*pifΔ*	T-7.1	15	6.6	(4.5 - 9.6)	21495.8	(3385.8 - 37341.3)	3246.4 (19.0)

[a] Depicts the position of the *URA3* reporter gene from the break site in kilobases. “T” represents telomeric side of the HO-break site. “C” refers to centromeric side of the HO-break site.

[c] GLU refers to glucose containing media. HO-endonuclease not expressed, thus representing No-break conditions.

[d] GAL refers to 2% galactose containing media. Galactose induces the expression of HO-endonuclease, thus generating double strand breaks (DSBs).

[e] Fold represents the increase in mutation frequency after “GAL” over “GLU” control. The numbers in parentheses indicate the mutation frequency relative to that in the no-homology strain.

[f] 2h induction of HO-endonuclease in 2% galactose containing media and plated on YEPD.
