# Supplementary material for: Microhomology-mediated end joining induces hypermutagenesis at breakpoint junctions
Source: PLoS Genet. 2017 Apr 18;13(4):e1006714. doi: 10.1371/journal.pgen.1006714 (PMC5413072; doi:10.1371/journal.pgen.1006714)
Supplement: S7 Table — a Depicts the position of the URA3 reporter gene from the break site in kilobases. “T” represents telomeric side of the HO-break site. “C” refers to centromeric side of the HO-break site. b Depicts the size of homology flanking the HO-cleavage site. c GLU refers to glucose containing media. HO-endonuclease not expressed, thus representing no-break conditions. d GAL refers to 2% galactose containing media. Galactose induces the expression of HO-endonuclease, thus generating double strand breaks (DSBs). e Fold represents the increase in mutation frequency “GAL” over “GLU” control. The numbers in parentheses indicate the mutation frequency relative to that in the no-homology strain. f 2 h induction of HO-endonuclease in 2% galactose containing media. (PDF) [file pgen.1006714.s018.pdf]

Table S7 Median frequencies of *can1* mutants (CAN^R) and 95% Confidence Interval (95% CI) with 20 J/m^2 U.V treatment were calculated by Fluctuation Analysis Calculator (FALCOR).

Strain	Genotype	Position (kb)[a]	Size of homology (bp)[b]	Frequency of CAN^R mutants ($X10^{-7}$) 20J U.V				
				GLU[c]		GAL[d]		
				MEDIAN	95% CI-range	MEDIAN	95% CI-range	Fold[e]
SS1		T- 7.1	0	56.4	(45.5 - 75.0)	164.3	(85.0 - 288.5)[f]	2.9 (1.00)
SS1		T- 7.1	0	63.8	(43.5 - 76.0)	346.5	(223.0 - 498.5)	5.4 (1.86)
SS2		T-7.1	203	22.6	(16.1 - 43.3)	173.2	(103.8 - 249.1)	7.7 (2.64)
SS3		T-11.5	203	31.8	(27.8 - 33.9)	259.9	(84.9 - 290.6)	8.2 (2.82)
SS4		T-7.1	15	10.5	(6.7 - 115.5)	671.4	(531.3 - 1058.8)	63.9 (22.04)
SS5		T-9.1	15	46.7	(26.8 - 172.8)	689.7	(483.9 - 895.5)	14.8 (5.09)
SS6		T-11.5	15	66.8	(63.6 - 90.7)	324.8	(237.4 - 475.8)	4.9 (1.68)
SS7		T-14.5	15	38.3	(31.0 - 56.5)	198.5	(66.2 - 279.3)	5.2 (1.79)
SS8		C-5.8	15	43.3	(12.6 - 107.5)	425.5	(284.6 - 1190.5)	9.8 (3.39)
SS9		C-7.2	15	131.5	(58.5 - 483.3)	220.0	(76.3 - 643.6)	1.7 (0.58)
SS10		C-20	15	53.0	(35.5 - 76.3)	373.1	(294.6 - 475.0)	7.0 (2.43)
SS11	*rev3Δ*	T-7.1	15	5.1	(3.6 - 8.6)	87.0	(21.4 - 187.1)	17.0 (5.85)
SS12	*rev1Δ*	T-7.1	15	12.3	(4.0 - 19.6)	64.0	(47.5 - 86.7)	5.2 (1.79)
SS13	*rad30Δ*	T-7.1	15	73.4	(45.8 - 119.8)	508.8	(394.0 - 778.0)	6.9 (2.39)
SS14	*rev3Δ* *rev1Δ* *rad30Δ*	T-7.1	15	7.4	(3.6 - 26.8)	77.8	(43.5 - 120.5)	10.5 (3.62)
SS15	*sgs1Δ*	T-7.1	15	93.3	(28.6 - 193.5)	10.4	(8.4 - 41.0)	0.1 (0.04)
SS16	*exo1Δ*	T-7.1	15	208.0	(109.0 - 286.5)	99.1	(80.9 - 237.5)	0.5 (0.16)
SS17	*pifΔ*	T-7.1	15	75.7	(27.9 - 121.9)	589.4	(151.9 - 1903.3)	7.8 (2.68)

[a] Depicts the position of the *URA3* reporter gene from the break site in kilobases. “T” represents telomeric side of the HO-break site. “C” refers to centromeric side of the HO-break site.

[b] Depicts the size of homology flanking the HO-cleavage site.

[c] GLU refers to glucose containing media. HO-endonuclease not expressed, thus representing No-break conditions.

[d] GAL refers to 2% galactose containing media. Galactose induces the expression of HO-endonuclease, thus generating double strand breaks (DSBs).

[e] Fold represents the increase in mutation frequency after “GAL” over “GLU” control. The numbers in parentheses indicate the mutation frequency relative to that in the no-homology strain.

[f] 2h induction of HO-endonuclease in 2% galactose containing media and plated on YEPD.
