# Supplementary material for: Microhomology-mediated end joining induces hypermutagenesis at breakpoint junctions
Source: PLoS Genet. 2017 Apr 18;13(4):e1006714. doi: 10.1371/journal.pgen.1006714 (PMC5413072; doi:10.1371/journal.pgen.1006714)
Supplement: S8 Table — The reporter is located at the 7.1 kb telomere-proximal location. a Mutations identified by sequencing of FOAR colonies with 20 J/m2 UV treatment. b GLU refers to glucose containing media. c GAL refers to galactose containing media. bp, base pairs; Pyr:Pur, ratio between Pyrimidine vs Purine mutations; In-Del, insertions and deletions. (PDF) [file pgen.1006714.s019.pdf]

Table S8 Analysis of *ura3* mutation events from FOA[R] survivors upon HO expression and UV irradiation. The reporter is located at the **7.1 kb** telomere proximal location

WT (Base in unresected strand)	Mutant base[a]	20J U.V		
		GLU[b]	GAL[c]	
		15bp	15bp	203 bp
A	G	1 (2.9%)	1 (1.9%)	5 (11.6%)
A	T	2 (5.8%)	3 (5.7%)	5 (11.6%)
A	C		1 (1.9%)	
Total A		3 (8.8%)	5 (9.6%)	10 (23.2%)
G	A	2 (5.8%)		
G	C	2 (5.8%)		1 (2.3%)
G	T	4 (11.7%)	1 (1.9%)	1 (2.3%)
Total G		8 (23.5%)	1 (1.9%)	2 (4.6%)
T	C		3 (5.7%)	2 (4.6%)
T	A	2 (5.8%)	5 (9.6%)	7 (16.2%)
T	G		2 (3.8%)	
Total T		2 (5.8%)	10 (19.2%)	9 (20.9%)
C	T	2 (5.8%)	4 (7.6%)	3 (6.9%)
C	G	3 (8.8%)		1 (2.3%)
C	A	7 (20.5%)	8 (15.3%)	1 (2.3%)
Total C		12 (35.2%)	12 (23.0%)	5 (11.6%)
Transition		5 (14.7%)	8 (15.3%)	10 (23.2%)
Tranversion		20 (58.8%)	20 (38.4%)	16 (37.2%)
In/Del		9 (26.4%)	23 (44.2%)	17 (39.5%)
Complex mutations		NA	1 (1.9%)	NA
Total mutations		34	52	43
Total Sequenced		34	52	43
Pyr:Pur		14:11	22:6	14:12

[a] Mutations identified by sequencing of FOA^R colonies with 20 J/m^2 U.V treatment
[b] GLU refers to glucose containing media.
[c] GAL refers to galactose containing media.
NA Not Available- No events found

bp, base pairs; Pyr:Pur , ratio between Pyrimidine vs Purine mutations; In-Del, insertions and deletions
