# Supplementary material for: Microhomology-mediated end joining induces hypermutagenesis at breakpoint junctions
Source: PLoS Genet. 2017 Apr 18;13(4):e1006714. doi: 10.1371/journal.pgen.1006714 (PMC5413072; doi:10.1371/journal.pgen.1006714)
Supplement: S9 Table — The reporter is located at the 7.1 kb telomere-proximal location. a Mutations identified by sequencing of FOAR events in a strain carrying two HO cleavage sites on two different chromosomes after HO expression. b GAL refers to galactose containing media. bp, base pairs; HYGR, hygromycin resistant; HYGS, hygromycin sensitive; Pyr:Pur, ratio between Pyrimidine vs Purine mutations; In-Del, insertions and deletions. (PDF) [file pgen.1006714.s020.pdf]

Table S9 Analysis of *ura3* mutation events from FOA[R] survivors upon two simultaneous HO cleavage events. The reporter is located at the **7.1 kb** telomere proximal location

WT (Base in unresected strand)	Mutant base[a]	GAL[b]	
		HYG[R]	HYG[S]
A	G	4 (7.1%)	2 (2.8%)
A	T	2 (3.5%)	7 (10.0%)
A	C	4 (7.1%)	2 (2.8%)
Total A		10 (17.8%)	11 (15.7%)
G	A	5 (8.9%)	5 (7.1%)
G	C	6 (10.7%)	1 (1.4%)
G	T	4 (7.1%)	17 (24.2%)
Total G		15 (26.7%)	23 (32.8%)
T	C		
T	A	2 (3.5%)	4 (5.7%)
T	G	3 (5.3%)	2 (2.8%)
Total T		5 (8.9%)	6 (8.5%)
C	T	1 (1.7%)	4 (5.7%)
C	G	2 (3.5%)	2 (2.8%)
C	A	14 (25.0%)	3 (4.2%)
Total C		17 (30.3%)	9 (12.8%)
Transition		10 (17.8%)	11 (15.7%)
Transversion		37 (66.0%)	38 (54.2%)
In/Del		9 (16.0%)	21 (28.5%)
Complex Mutations		NA	NA
Total Mutations		56	70
Total Sequenced		56	70
Pyr:Pur		22:25	15:34

[a] Mutations identified by sequencing of FOA^R events in a strain carrying two HO cleavage sites on two different chromosomes after HO expression.
[b] GAL refers to galactose containing media.
NA Not Available- No events found

bp, base pairs; $HYG^{R,}$ hygromycin resistant; HYG^S, hygromycin sensitive; Pyr:Pur, ratio between Pyrimidine vs Purine mutations; In-Del, insertions and deletions
