# Supplementary material for: Microhomology-mediated end joining induces hypermutagenesis at breakpoint junctions
Source: PLoS Genet. 2017 Apr 18;13(4):e1006714. doi: 10.1371/journal.pgen.1006714 (PMC5413072; doi:10.1371/journal.pgen.1006714)
Supplement: S10 Table — (PDF) [file pgen.1006714.s021.pdf]

Table S10 Strain List

Strain	Position (kb)[a]	Size of homology (bp)[b]	Genotype	References
JKM139	NA	0	*hoΔ MAT**a** hmlΔ::ADE1 hmrΔ::ADE1 ade1-100 leu2-3,112 lys5 trp1::hisG ura3-52 ade3::GAL-HO*	(Ma, Kim et al. 2003)
JKM179	NA	0	*hoΔ MAT**α** hmlΔ::ADE1 hmrΔ::ADE1 ade1-100 leu2-3,112 lys5 trp1::hisG ura3-52 ade3::GAL-HO*	(Ma, Kim et al. 2003)
YDV501A	NA	0	*JKM179 ura3Δ0*	(Villarreal, Lee et al. 2012)
M18	NA	18	*JKM179 MAT**α**Z1:: 18 bp microhomology ::HPH*	This Study
M18-7	NA	18	*MH18 URA3 on ChrIII at Telomeric 7.1 kb position*	This Study
YDV60.18	NA	18	*JKM139 MAT**a**Z1:: 18 bp microhomology ::HPH*	(Villarreal, Lee et al. 2012)
MH15	NA	15	*YDV501A MAT**α**Z1:: 15 bp microhomology ::HPH*	This Study
SS4	T-7.1	15	*MH15 URA3 on ChrIII at Telomeric 7.1 kb position*	This Study
SS5	T-9.1	15	*MH15 URA3 on ChrIII at Telomeric 9.1 kb position*	This Study
SS6	T-11.5	15	*MH15 URA3 on ChrIII at Telomeric 11.5 kb position*	This Study
SS7	T-14.5	15	*MH15 URA3 on ChrIII at Telomeric 14.5 kb position*	This Study
SS8	C-5.8	15	*MH15 URA3 on ChrIII at Centromeric 5.8 kb position*	This Study
SS9	C-7.2	15	*MH15 URA3 on ChrIII at Centromeric 7.2 kb position*	This Study
SS10	C-20	15	*MH15 URA3 on ChrIII at Centromeric 20,kb position*	This Study
SS11	T-7.1	15	*SS4 rev3Δ::LEU2*	This Study
SS12	T-7.1	15	*SS4 rev1Δ::NAT*	This Study
SS13	T-7.1	15	*SS4 rad30Δ::KAN*	This Study
SS14	T-7.1	15	*SS4 rev3Δ::LEU2 rev1Δ::NATrad30Δ::KAN*	This Study
SS15	T-7.1	15	*SS4 sgs1Δ::KAN*	This Study
SS16	T-7.1	15	*SS4 exo1Δ::KAN*	This Study
SS17	T-7.1	15	*SS4 pif1Δ::KAN*	This Study

Strain	Position (kb)[a]	Size of homology (bp)[b]	Genotype	References
SS18	T-7.1	15	*SS4 HO cut site Δ*	This Study
SS203	NA	203	*JKM179 MAT**α**Z1:: 203 bp homology::HPH*	This Study
SS2	T-7.1	203	*SS203 URA3 on ChrIII URA3 at Telomeric 7.1 kb position*	This Study
SS3	T-11.5	203	*SS203 URA3 on ChrIII URA3 at Telomeric 11.5 kb position*	This Study
SS1	T-7.1	0	*JKM179 MAT**α**Z1::HPH::URA3 on ChrIII URA3 at Telomeric 7.1 kb position*	This Study
SS1-2	T-7.1	0	*SS1 lig4Δ::KAN*	This Study
SS527	NA	527	*JKM179 MATαZ1:: 527 bp homology::HYG on ChrIII*	This Study
YDV501B	NA	17	*JKM179 ura3-52::MAT**a**-cut-site URA3 AmpR 17 bp microhomology to MATα and MAT**a***	This Study
YDV501.17	NA	17	*YDV501B MATαZ1::HPH::17 bp microhomology to MAT**a** and MATα*	This Study
SS17INTER	NA	17	*YDV501.17 URA3::TRP1*	(Villarreal, Lee et al. 012)
SS17INTER.7.1	T-7.1	17	*SS17INTER URA3 on ChrIII at Telomeric 7.1 kb position*	This Study
18-14T15A	NA	18	*JKM179 MAT**a**Z1::HPH::18 bp microhomology with mismatch at 14 bp and 15 bp position*	This Study
18-3A4A	NA	18	*JKM179 MAT**a**Z1::HPH::18bp microhomology with mismatch at 3bp and 4bp position*	This Study
18-9G	NA	18	*JKM179 MAT**a**Z1::HPH::18 bp microhomology with mismatch at 9 bp position*	This Study
18-10C11C	NA	18	*JKM179 MAT**a**Z1::HPH::18 bp microhomology with mismatch at 10 bp and 11 bp position*	This Study
18-16T	NA	18	*JKM179 MAT**a**Z1::HPH::18 bp microhomology with mismatch at 16 bp position*	This Study
18-9-T	NA	18	*JKM179 MAT**a**Z1::HPH::18 bp microhomology with mismatch at 9 bp position*	This Study
18-3A	NA	18	*JKM179 MAT**a**Z1::HPH::18 bp microhomology with mismatch at 3 bp position*	This Study
18-5G	NA	18	*JKM179 MAT**a**Z1::HPH::18 bp microhomology with mismatch at 5 bp position*	This Study
18-3A15A	NA	18	*JKM179 MAT**a**Z1::HPH::18 bp microhomology with mismatch at 3 bp and 15 bp position*	This Study
MH18-BrdU	NA	18	*MH18 BrdU-inc::TRP1*	This Study
SS527-BrdU	NA	527	*SS527 BrdU-inc::TRP1*	This Study
SS203-BrdU	NA	203	*SS203 BrdU-inc::TRP1*	This Study

[a] Depicts the position of the *URA3* reporter gene from the break site in kilobases. “T” represents telomeric side of the HO-break site. “C” refers to centromeric side of the HO-break site.

[b] Depicts the size of homology flanking the HO-cleavage site.

NA Not Applicable
