# Supplementary material for: Microhomology-mediated end joining induces hypermutagenesis at breakpoint junctions
Source: PLoS Genet. 2017 Apr 18;13(4):e1006714. doi: 10.1371/journal.pgen.1006714 (PMC5413072; doi:10.1371/journal.pgen.1006714)
Supplement: S24 Table — a represents the ratio of yeast cells with a wild-type URA3 gene and cells with mutations in ura3 (both with mutated HO cleavage site at the MAT locus). b represents the average frequency of FOAR cells upon direct plating on FOA-GAL. c represents the median FOAR frequency and 95% Confidence Interval of cells plated on YEP-GAL followed by replica plating. d,e Median frequencies of ura3 mutants (FOAR) and 95% Confidence Interval (95% CI) were calculated by Fluctuation Analysis Calculator (FALCOR). (PDF) [file pgen.1006714.s035.pdf]

Table S24- Efficiency of Replica Plating

Ratio of URA+ versus FOA resistant (FOA^r) cells[a], 10^5:1

Cells Plated	Repeat of Experiment	Direct Plating, FOA^r frequency (X10^{-5})[b]	Replica Plating[c] (X10^{-5})		Efficiency of Replica Plating (%)
			Median[d]	95% CI-range[e]	
10^7	1	1.12	0.4	0.3 - 0.8	44.1
	2	1.16	0.5	0.3 - 0.7	46.3
	3	1.38	0.5	0.4 - 1.2	40.6

Ratio of URA+ versus FOA resistant (FOA^r) cells[a], 10:1

Cells Plated	Repeat of Experiment	Direct Plating, FOA^r frequency (X10^{-1})[b]	Replica Plating[c] (X10^{-1})		Efficiency of Replica Plating (%)
			Median[d]	95% CI-range[e]	
10^3	1	1.1	0.7	0.6 - 0.9	66.3
	2	0.8	0.4	0.3 - 0.6	45.0
	3	1.2	0.5	0.4 - 1.1	39.9

[a] represents the ratio of yeast cells with a wild-type *URA3* gene and cells with mutations in *ura3* (both with mutated HO cleavage site at the *MAT* locus)
[b] represents the average frequency of FOA^R cells upon direct plating on FOA-GAL
[c] represents the median FOA^R frequency and 95% Confidence Interval of cells plated on YEP-GAL followed by replica plating
[d,e] Median frequencies of *ura3* mutants (FOA^R) and 95% Confidence Interval (95% CI) were calculated by Fluctuation Analysis Calculator (FALCOR).
